# Synthesis and Biological Evaluation of a Marine Steroidal Alkaloid Analog (3*S*)-Peniadenisteroid A

**DOI:** 10.3390/md24100352

**Published:** 2026-10-06

**Authors:** Xuetao Zhang, Xiangyu Wang, Mireguli Maimaitiming, Jichen Yang, Jie Wang, Danling Gao, Lujia Yang, Xinxin Zhang, Zhiqing Liu, Pingyuan Wang, Chang-Yun Wang

**Affiliations:** 1 Key Laboratory of Marine Drugs and Key Laboratory of Evolution and Marine Biodiversity (Ministry of Education), School of Medicine and Pharmacy, Institute of Evolution & Marine Biodiversity, Ocean University of China, Qingdao 266003, China; xuetaozhang@stu.ouc.edu.cn (X.Z.); wxy4336@stu.ouc.edu.cn (X.W.); mire@xjmu.edu.cn (M.M.); yangjichen@stu.ouc.edu.cn (J.Y.); wj2797@stu.ouc.edu.cn (J.W.); wugulan123@163.com (D.G.); yanglujia_0407@163.com (L.Y.); 2 Department of Forensic Medicine, College of Basic Medical Sciences, Xinjiang Medical University, Urumqi 830011, China; 3 Marine Biomedical Research Institute of Qingdao, School of Medicine and Pharmacy, Ocean University of China, Qingdao 266003, China; xxzhang.2012@qdmbri.com; 4 Laboratory for Marine Drugs and Bioproducts, Qingdao Marine Science and Technology Center, Qingdao 266237, China

**Keywords:** peniadenisteroid A, striasteroid C, (3*S*)-peniadenisteroid A, steroidal alkaloid, synthesis, anti-inflammatory, network pharmacology

## Abstract

Peniadenisteroid A, also known as striasteroid C, is a unique marine-derived natural steroidal alkaloid with a characteristic adenine–steroid hybrid scaffold, in which an adenine moiety is bound to the C-3 position of the steroid skeleton. This natural metabolite displays evident antiviral activity through neuraminidase inhibition, with an IC_50_ value of 6.15 μM. In this work, the C-3 epimer, (3*S*)-peniadenisteroid A, was successfully synthesized in eight steps with an overall yield of 3.9%. Biological screening results revealed that the intermediate (3*S*)-**P6** exhibited prominent inhibitory activity against cellular nitric oxide (NO) production, while both (3*S*)-peniadenisteroid A and peniadenisteroid A presented weak anti-inflammatory effects. Further mechanistic studies indicated that (3*S*)-**P6** exerts significant anti-inflammatory bioactivity by negatively regulating the STAT3/NF-κB and HIF-1α signaling pathways. Moreover, network pharmacology combined with molecular docking predicted Heat Shock Protein 90α (HSP90α) as the putative binding target of (3*S*)-**P6**. Overall, this study completes the synthesis and biological evaluation of (3*S*)-peniadenisteroid A, highlighting that intermediate (3*S*)-**P6** is a valuable lead compound for further anti-inflammatory drug discovery.

## 1. Introduction

**Figure 1:**
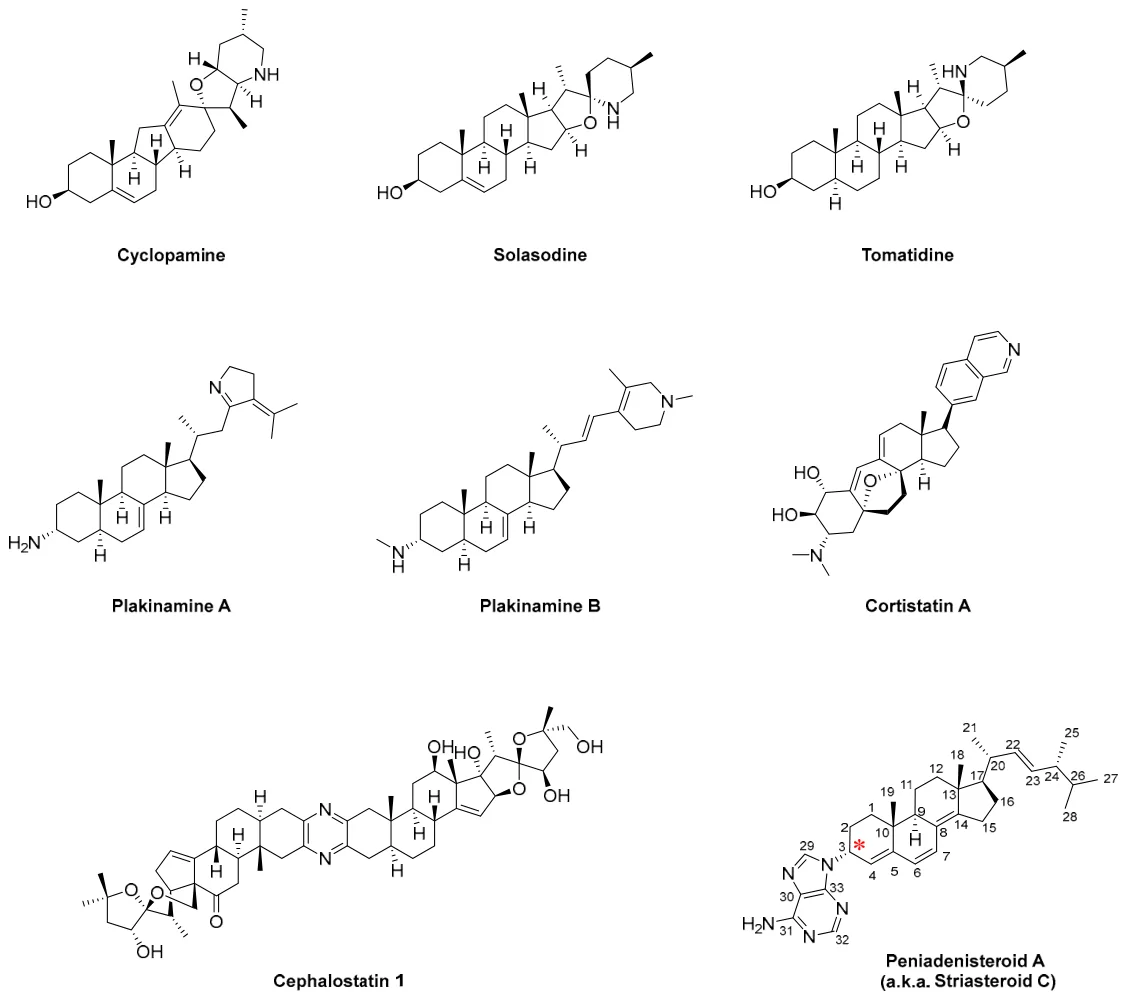
Chemical structures of representative steroidal alkaloid natural products. * Denotes a chiral center.

Steroidal alkaloids are an important class of secondary metabolites, predominantly distributed in plant families including Solanaceae, Buxaceae, Apocynaceae, and Liliaceae [1,2]. Structurally, they possess a fundamental steroidal skeleton integrated with a nitrogen atom, which is embedded either in the ring system or the side chain (Figure 1) [1]. Steroidal alkaloids and their derivatives, exemplified by cyclopamine, solasodine, and tomatidine (Figure 1), exhibit diverse pharmacological properties, including potent anticancer, anti-inflammatory, and antibacterial activities [1,2,3,4]. Given their unique structural features and versatile biological activities, steroidal alkaloids have garnered substantial research interest, especially in the exploration of marine natural products.

The first reported marine-derived steroidal alkaloids, plakinamine A and plakinamine B (Figure 1), were isolated from the marine sponge *Plakina* sp. by Rosser et al. in 1984 [5]. Subsequent biological tests demonstrated that plakinamine A suppressed the growth of *Staphylococcus aureus* and *Candida albicans* at doses of 25 μg/disk and 10 μg/disk, respectively. Meanwhile, the hydrochloride derivative of plakinamine B exerted inhibitory effects on the same pathogens at lower doses of 10 μg/disk and 2 μg/disk [5]. Furthermore, cortistatins represent the most extensively investigated subclass of marine-derived steroidal alkaloids. These compounds are characterized by a highly functionalized steroidal core with multiple chiral centers, which is conjugated to nitrogen-containing heterocyclic moieties such as isoquinoline. As illustrated in Figure 1, cortistatin A was first isolated from the marine sponge *Corticium simplex* by Aoki et al. in 2006 and it was reported with an anti-angiogenic activity against human umbilical vein endothelial cells (HUVECs), with an IC_50_ value of 1.8 nM [6]. In addition, Nicolaou et al. reported in 2009 that cortistatin A serves as a high-affinity ligand for the protein kinases Rho-associated coiled-coil containing protein kinase (ROCK), cyclin-dependent kinase 8 (CDK8), and cyclin-dependent kinase 11 (CDK11) [7]. Cephalostatin 1 (Figure 1), another representative steroidal alkaloid isolated from the marine worm *Cephalodiscus gilchristi*, possesses a unique bissteroidal skeleton and represents a structurally and biologically significant member of this compound class [8]. It exhibits exceptionally potent antitumor activity, with an ED_50_ value of less than 1 pM against P388 leukemia cells and potent cytotoxic activity against all 60 human tumor cell lines in the National Cancer Institute (NCI) screening panel [8,9,10].

Inspired by the novel chemical structures, diverse and excellent pharmacological activities, as well as high drug-likeness of steroidal alkaloids, we carried out research on the discovery and bioactivity evaluation of steroidal alkaloids from marine-derived microorganisms. Subsequently, a novel steroidal alkaloid, named peniadenisteroid A (Figure 1), was isolated and characterized from a deep-sea-sediment-derived fungus *Penicillium janthinellum* SH0301, and its structure was established by NMR, HRMS, and ECD analyses. Structurally, peniadenisteroid A consists of an ergostane-type steroid and a purine moiety, which are linked via a C-N bond between C-3 position of the steroid and N-9 position of the purine. The absolute configuration at C-3 was proposed to be *R*-configuration. Unfortunately, cytotoxic and anti-inflammatory activity screening revealed that it only exhibited weak inhibitory activity against NO production in LPS-induced RAW264.7 macrophages, with an inhibition rate of 43% at 20 μM. In 2021, Dong et al. isolated peniadenisteroid A from the mangrove sediment-derived fungus *Penicillium brefeldianum* ABC190807 [11]. Most recently, Zhou et al. reported the isolation of peniadenisteroid A from the marine-derived fungus *Striaticonidium cinctum* SCSIO41432 and named it striasteroid C. Notably, they unambiguously determined its absolute configuration for the first time via X-ray diffraction analysis, revealing an *R*-configuration at the C-3 stereo center. Meanwhile, they reported that peniadenisteroid A exhibited potent neuraminidase-inhibitory activity with an IC_50_ value of 6.15 μM [12].

Further investigations on peniadenisteroid A are hindered by its limited natural availability, due to its low abundance and complex extraction process. Furthermore, the effect of the C-3 chiral center on the biological activity of this compound remains to be elucidated, and no synthetic strategy for peniadenisteroid A or its diastereomers has been reported to date. Therefore, this study aims to explore the synthesis of peniadenisteroid A and its C-3 diastereomer, preliminarily compare the activity differences between analogs with opposite configurations at the C-3 position of the steroid nucleus, and to perform systematic bioactivity screening of structurally novel synthetic intermediates in order to identify lead candidates with therapeutic potential.

## 2. Results

### 2.1. Chemistry

**Figure 2:**
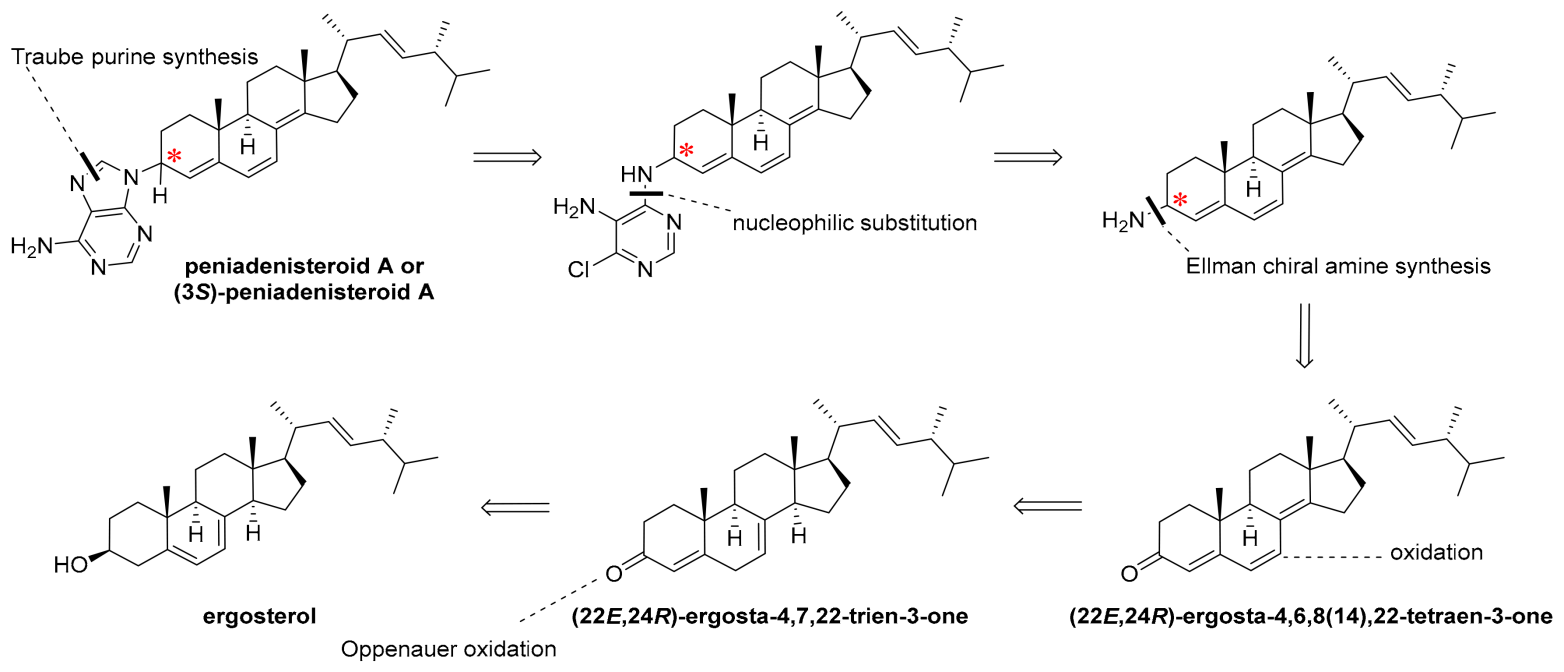
Retrosynthetic analysis of compound peniadenisteroid A and (3*S*)-peniadenisteroid A. * Denotes a chiral center.

Herein, a rational retrosynthetic analysis was performed for peniadenisteroid A and its C-3 epimer, (3*S*)-peniadenisteroid A (Figure 2). Both target compounds could be constructed via the Traube purine synthesis, in which the key chiral 4,5-diaminopyrimidine intermediate undergoes cyclization with trimethoxymethane. This chiral diaminopyrimidine intermediate would be available through a nucleophilic substitution reaction between 5-amino-4,6-dichloropyrimidine and a chiral steroidal amine precursor. The chiral steroidal amine could be synthesized from (22*E*,24*R*)-ergosta-4,6,8(14),22-tetraen-3-one by employing Ellman’s *tert*-butanesulfinamide-mediated asymmetric amine synthesis. (22*E*,24*R*)-ergosta-4,6,8(14),22-tetraen-3-one, in turn, could be prepared via DDQ oxidation of (22*E*,24*R*)-ergosta-4,7,22-trien-3-one, which would be ultimately derived from the oxidation of ergosterol.

**Scheme 1:**
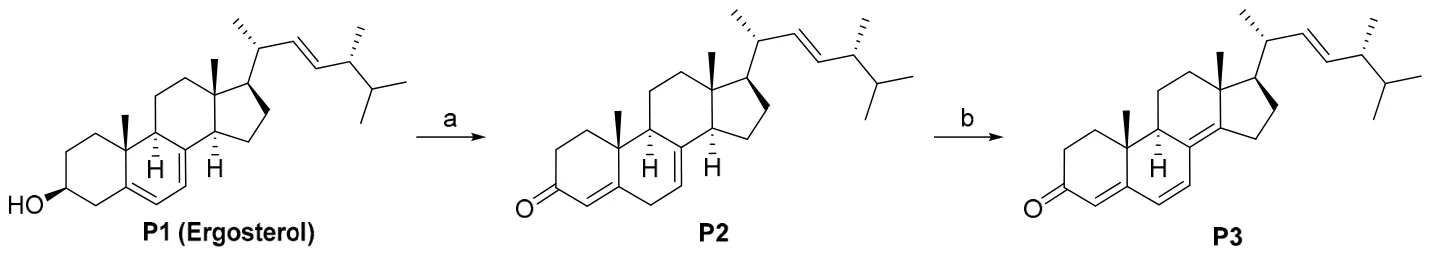
Synthesis of compound **P3**. Reagents and conditions: (**a**) aluminium isopropoxide, cyclohexanone, toluene, rt-115 °C, 3 h, 70%; (**b**) DDQ, 1,4-dioxane, 110 °C, 4 h, 39%.

Guided by reasonable retrosynthetic analysis, we performed synthetic studies of peniadenisteroid A and (3*S*)-peniadenisteroid A and the synthetic routes were summarized in Scheme 1. As shown in Scheme 1, compound (22*E*,24*R*)-ergosta-4,7,22-trien-3-one (**P2**) was efficiently prepared by oxidation of commercially available ergosterol (**P1**) in 70% yield via Oppenauer oxidation [13]. However, the subsequent oxidation of **P2** to **P3** turned out to be a challenging transformation. Initial attempts employing Jones reagent as the oxidant in THF at room temperature failed to obtain the desired product **P3** (Table 1, entry 1) [14,15]. Optimizations regarding reaction temperature, solvent, and oxidant equivalents were then conducted, yet none of these modifications enhanced the reaction efficiency (Table 1, entries 2–4). Further screening of alternative oxidants, including PCC, PDC, IBX, and DMP, also afforded no improvement in oxidation yields (Table 1, entries 5–12) [16,17,18,19]. Gratifyingly, the adoption of DDQ as the oxidant in 1,4-dioxane at 40 °C moderately promoted the transformation, furnishing **P3** in 19% yield, which was higher than that obtained under the room-temperature conditions (Table 1, entries 13–15). Graduated elevation of the reaction temperature to 110 °C combined with a shortened reaction duration of 4 h further improved the outcome, delivering **P3** with a yield of 39% (Table 1, entry 16). In contrast, prolonged reaction time led to a slight reduction in product yield (Table 1, entry 17). Ultimately, the optimal reaction conditions for the synthesis of **P3** are: DDQ as the oxidant, 1,4-dioxane as the solvent at 110 °C for 4 h. To our delight, when the reaction was scaled up to the 10 g scale, no decrease in yield was observed.

**Scheme 2:**
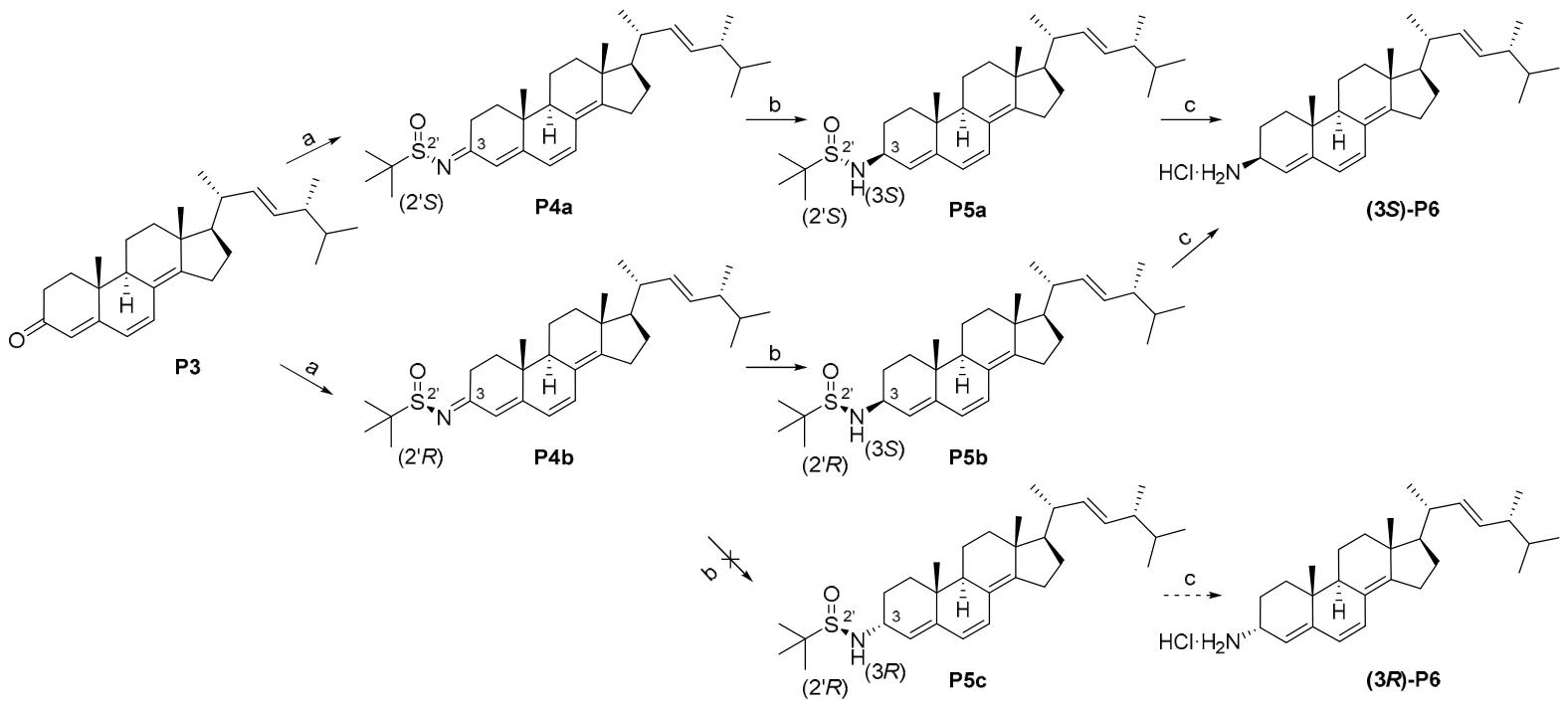
Synthesis of compound (3*S*)-**P6**. Reagents and conditions: (**a**) (*S*)-2-methylpropane-2-sulfinamide for **P4a** and (*R*)-2-methylpropane-2-sulfinamide for **P4b**, titanium tetraisopropanolate, THF, 70 °C, 24 h; (**b**) DIBAL-H, THF, −78 °C-rt, 5 h, 52% in two steps for **P5a** and 62% in two steps for **P5b**; (**c**) 2M HCl in 1,4-dioxane, rt, 4 h, 80%. Solid arrows: successful reactions; dotted arrows: planned but unattempted routes; crossed arrows: failed reactions.

**Figure 3:**
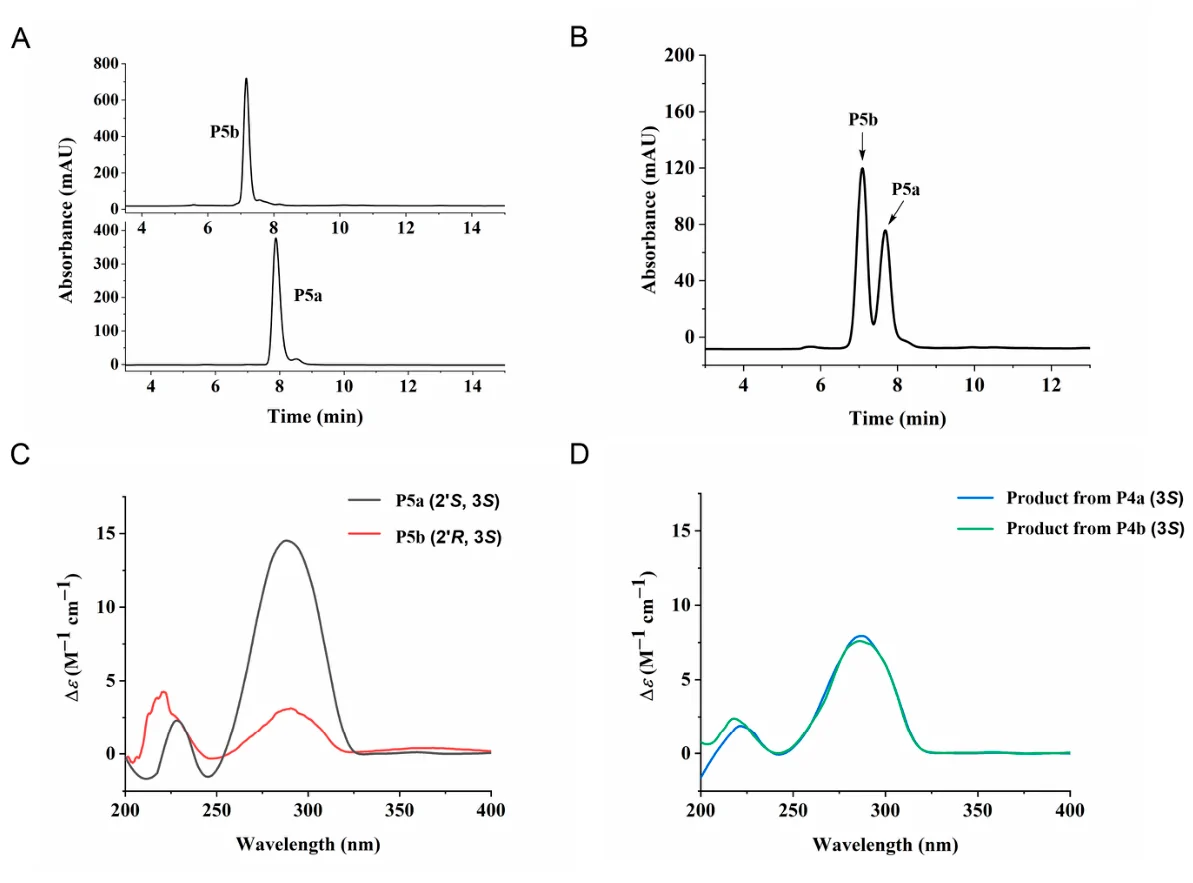
Absolute configuration studies of **P5a**, **P5b**, product from **P4a** and product from **P4b**. (**A**) Chiral HPLC profiles of compounds **P5a** and **P5b**. (**B**) Chiral HPLC analysis: **P5a** co-injected with **P5b**. (**C**) ECD spectral comparison of **P5a** and **P5b** for absolute configuration. (**D**) ECD spectral comparison of product from **P4a** and product from **P4b** for absolute configuration.

As depicted in Scheme 2, following the successful preparation of sufficient **P3** intermediate, we proceeded to construct the C-3 stereo center on the steroid scaffold. On the basis of the aforementioned retrosynthetic analysis, we sought to install the C-3 stereo center through chiral induction using Ellman’s *tert*-butanesulfinamide of different chirality to afford the target chiral steroidal amines [20,21]. Subsequently, intermediate **P3** was reacted with the (*S*)- and (*R*)-configured *tert*-butylsulfinimine to afford the corresponding imine intermediates **P4a** and **P4b**, respectively. Reduction of compound **P4a** with DIBAL-H, followed by deprotection of the *tert*-butanesulfinyl group, afforded the C-3 *S*-configured steroidal amine (3*S*)-**P6**. However, following the same procedure that was used to prepare compound (3*S*)-**P6** from compound **P4b** failed to obtain intermediate (3*R*)-**P6**. By comparing the reaction product of **P5a**, (3*S*)-**P6**, with that of **P5b**, we conclude that the reduction-deprotection product of **P4b** is (3*S*)-**P6** rather than (3*R*)-**P6** (Scheme 2 and Figure 3D). We speculate that the C-3 *R*-configured steroidal amine intermediate was not successfully synthesized. Accordingly, we performed a systematic analysis of the reduction product **P5a/b** derived from intermediate **P4a/b** using mass spectrometry, NMR, HPLC and ECD spectroscopy.

As shown in Figures S5–S8, S20 and S21, compounds **P5a** and **P5b** showed identical molecular weights (*m*/*z* (ESI) [M + H]^+^ = 498) and nearly identical NMR data. The HPLC analysis revealed that compounds **P5a** and **P5b** exhibited similar retention times (Figure 3A,B). Additionally, if the two compounds indeed vary at two stereogenic centers, their ECD profiles should not exhibit such minor differences; nevertheless, the measured ECD differences were considerably smaller than expected. These unexpectedly small ECD differences led us to suspect that the two compounds may in fact differ at only a single stereogenic center. (Figure 3C). Further 2D NOESY experiments on **P5a** and **P5b** showed no NOE cross-peak between H-3 and the C-19 angular methyl protons in either compound, suggesting that both share the same C-3 stereochemistry and are likely of the *S* configuration (Figures S22 and S23). Taken together, we concluded that reduction of **P4b** with DIBAL-H led to compound **P5b** rather than expected compound **P5c** (Scheme 2). The DIBAL-H reduction of the imine appears to be governed primarily by the pre-existing stereochemistry of the steroid skeleton rather than by the sulfur configuration of the Ellman auxiliary; this accounts for both **P4a** and **P4b** furnishing the same (3*S*) amine. Herein, although we failed to obtain (3*R*)-**P6**, (3S)-**P6** was successfully synthesized, enabling us to proceed with the synthesis of (3*S*)-peniadenisteroid A following the aforementioned retrosynthetic analysis.

**Scheme 3:**
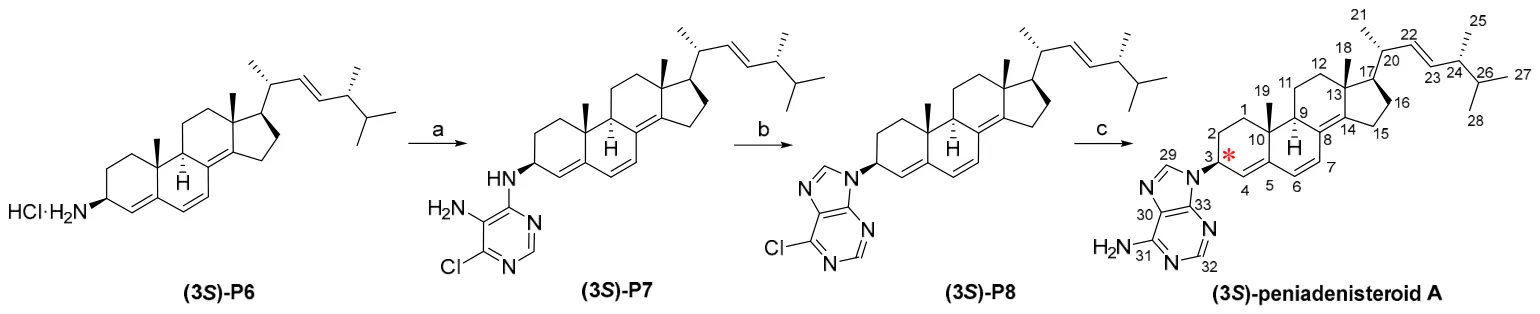
Semi-synthesis of (3*S*)-peniadenisteroid A. Reagents and conditions: (**a**) 5-amino-4,6-dichloropyrimidine, NaHCO_3_, *n*-butanol, 118 °C, 24 h, 66%; (**b**) trimethoxymethane, conc. HCl, rt, 6 h, 64%; (**c**) ammonia (7M in MeOH), THF, 70 °C, 30 h, 69%. * Denotes a chiral center.

Subsequently, we initiated the synthesis of (3*S*)-peniadenisteroid A, and the results are illustrated in Scheme 3. Compound (3*S*)-**P6** was reacted with NaHCO_3_, followed by nucleophilic substitution with 5-amino-4,6-dichloropyrimidine to furnish intermediate (3*S*)-**P7** in 66% yield. Acid-catalyzed intramolecular cyclization of (3*S*)-**P7** using trimethoxymethane then afforded the cyclized intermediate (3*S*)-**P8** [22]. Finally, treatment of (3*S*)-**P8** with 7 M ammonia in methanol delivered the target product (3*S*)-peniadenisteroid A in 69% yield.

### 2.2. Characteristic Analysis

The high-resolution electron spray ionization mass (HRMS-ESI) of compound (3*S*)-peniadenisteroid A exhibited [M + H]^+^ peak at *m*/*z* 512.3751 (C_33_H_46_N_5,_ calcd for 512.3748) (Figure S19) corresponding to the reported molecular formula of compound striasteroid C [12]. Whereas the ^1^H and ^13^C NMR data of natural striasteroid C and our isolated peniadenisteroid A were in close agreement, the synthesized epimer (3*S*)-peniadenisteroid A exhibited substantial discrepancies from both. As shown in Table 2, the ^1^H NMR spectrum of peniadenisteroid A recorded in CDCl_3_ exhibited the C-3 methine proton at *δ*_H_ 5.31 (t, *J* = 5.1 Hz) and the ^13^C NMR signal at *δ*_C_ 49.0 ppm, which were essentially identical to the values reported for striasteroid C (*δ*_H_ 5.32, t, *J* = 5.0 Hz; *δ*_C_ 49.1 ppm). However, (3*S*)-peniadenisteroid A showed different chemical shifts around the C-3 stereocenter, with H-3 observed at *δ*_H_ 5.37 (t, *J* = 5.0 Hz) and C-3 at *δ*_C_ 52.0 ppm. The neighboring H-4 signal also shifted upfield to *δ*_H_ 5.44 (d, *J* = 4.8 Hz), compared with *δ*_H_ 5.56 (d, *J* = 4.9 Hz) for peniadenisteroid A, and the C-4 carbon signal moved downfield from *δ*_C_ 119.9 to *δ*_C_ 116.8 ppm. Divergences were further noted at C-1 (*δ*_C_ 33.2 vs. 29.6), C-2 (*δ*_C_ 27.9 vs. 25.7). (Table 2, Figures S15–S18). These differences in the C-3/H-3 region and adjacent carbons are suggestive of an epimeric relationship between the synthesized compound and the natural product, an assignment that will be further substantiated by ECD, chiral-phase HPLC, and single-crystal X-ray analyses.

**Figure 4:**
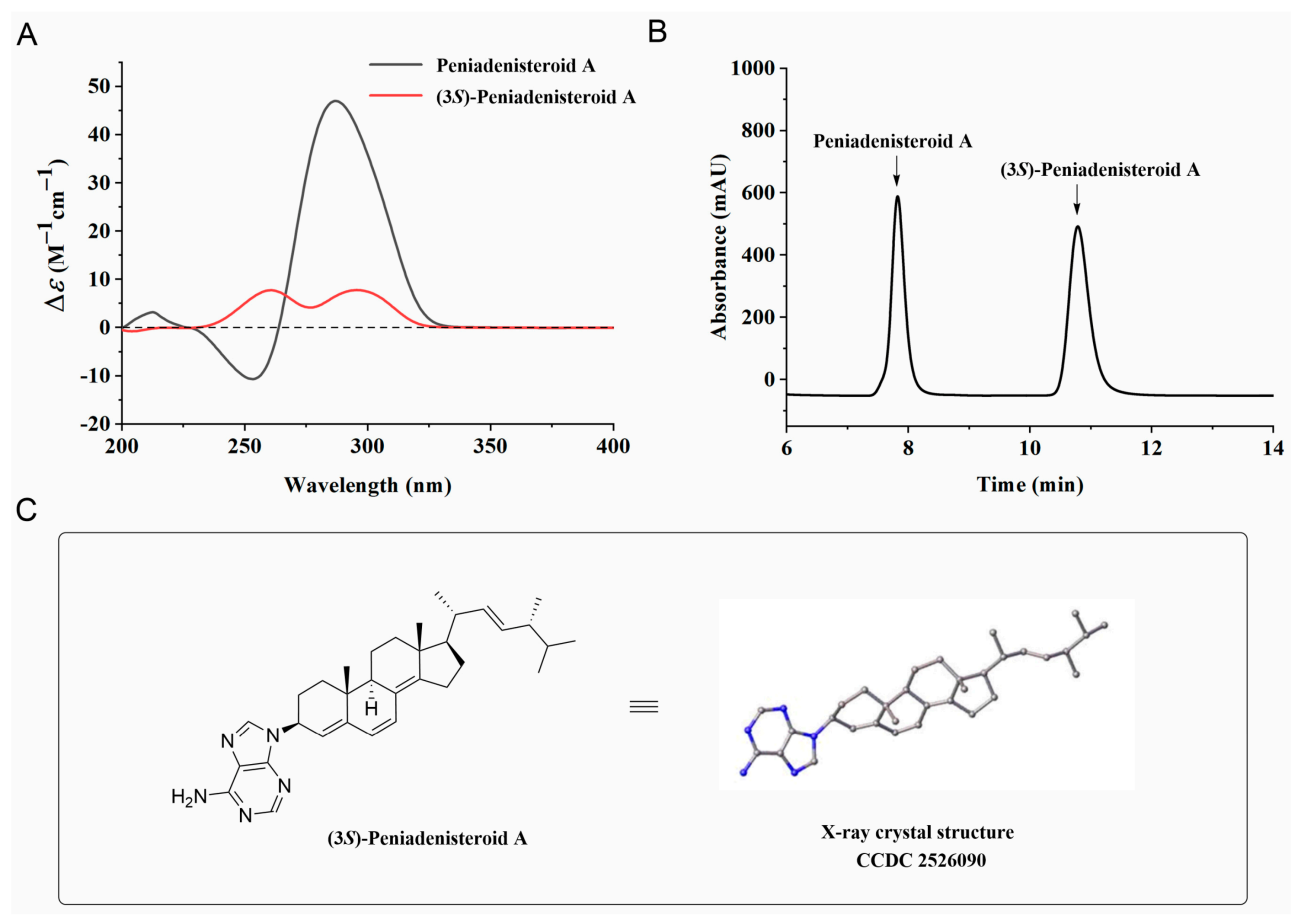
Absolute configuration studies of (3*S*)-peniadenisteroid A. (**A**) ECD spectral comparison for absolute configuration. (**B**) Chiral HPLC analysis: (3*S*)-peniadenisteroid A co-injected with peniadenisteroid A. (**C**) Crystal structure of (3*S*)-peniadenisteroid A determined by X-ray diffraction (CCDC number: 2526090).

Analysis of the ECD spectra indicated that peniadenisteroid A exhibited a negative Cotton effect at ~250 nm and a positive one at ~300 nm (Figure 4A). By comparison, (3*S*)-peniadenisteroid A displayed only weak positive Cotton effect signals at both wavelengths, with an opposite sign at 250 nm and a substantially diminished intensity at 300 nm. This clearly distinguishes the synthetic epimer from the natural product and is consistent with an inverted C-3 configuration. Meanwhile, chiral HPLC analysis showed that peniadenisteroid A displayed a retention time of 7.8 min, whereas (3*S*)-peniadenisteroid A eluted at 10.8 min (Figure 4B). Furthermore, the structure of (3*S*)-peniadenisteroid A was unambiguously confirmed by single-crystal X-ray diffraction analysis (Figure 4C). In summary, we have accomplished the synthesis of (3*S*)-peniadenisteroid A via an 8-step sequence with an overall yield of 3.9%. While the natural product itself remains synthetically inaccessible, its C-3 epimer, (3*S*)-peniadenisteroid A, was successfully prepared, offering useful guidance for the future synthesis of the natural product.

### 2.3. Biological Evaluation

(3*S*)-Peniadenisteroid A and its synthetic intermediates were evaluated for their anti-inflammatory activity by measuring NO production in LPS-stimulated RAW264.7 macrophages. As listed in Table 3, both (3*S*)-peniadenisteroid A and peniadenisteroid A showed weak inhibitory activity against LPS-induced NO production. Notably, intermediates **P3** and (3*S*)-**P6** demonstrated promising NO production inhibitory activities, with inhibition rates reaching 59.7% and 86.1% at a concentration of 10 μM, respectively. To further elucidate the anti-inflammatory mechanism and molecular target of (3*S*)-**P6**, a high-throughput screening assay was performed to evaluate its regulatory effects on multiple signaling pathways, including STAT3/NF-κB, HIF-1α, WNT/β-catenin, and p53. The results revealed that (3*S*)-**P6** exerted prominent inhibitory activity against two core transcription factors, STAT3/NF-κB and HIF-1α (Table 4).

**Figure 5:**
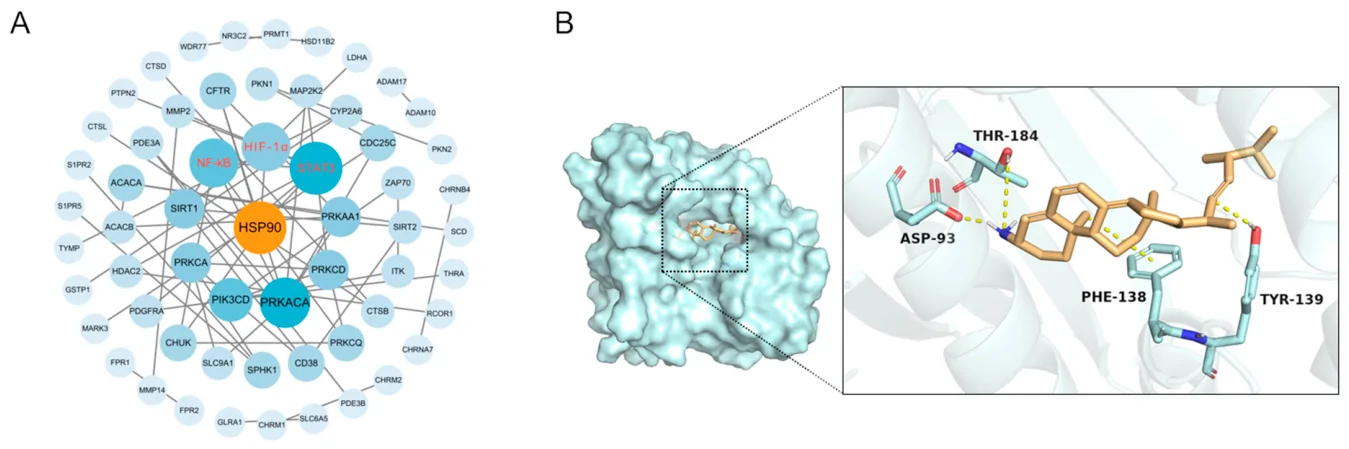
Network pharmacology and molecular docking analysis of compound (3*S*)-**P6**. (**A**) Prediction of potential targets for (3*S*)-**P6** via Network Pharmacology. GeneCards database (https://www.genecards.org, accessed on 31 January 2026); SuperPRED (https://prediction.charite.de, accessed on 31 January 2026); STRING (https://cn.string-db.org, accessed on 31 January 2026). Red font denotes signaling pathway results consistent with Table 4 (Section 2.3). (**B**) Docking-predicted binding model for (3*S*)-**P6** within HSP90α (PDB code: 1UY6).

Given the anti-inflammatory potential of compound (3*S*)-**P6** and its inhibitory effects on the HIF-1α and STAT3/NF-κB signaling pathways, we conducted a preliminary exploration of its possible targets under the guidance of network pharmacology. First, using “inflammation” as a key term, we searched the GeneCards database for disease-related therapeutic targets and obtained 12,121 candidate targets. Subsequently, based on the chemical structure of (3*S*)-**P6**, we queried the SuperPRED database to predict its potential binding targets. A total of 149 potential binding targets were compiled. These two sets, the disease-related targets (12,121) and the compound-predicted targets (149), were then intersected using a Venn diagram, yielding 141 overlapping targets (Figure S24). Following this initial screening, a protein–protein interaction (PPI) network was constructed using the STRING database. The resulting data were processed and visualized with Cytoscape software (version 3.10.0), and nodes were ranked according to their degree centrality. PPI network analysis revealed 10 proteins that may be closely associated with the anti-inflammatory activity of (3*S*)-**P6** (Figure 5A). It is noteworthy that HIF-1α, STAT3 and NF-κB were among the 10 most relevant targets identified, which had already been identified in our earlier high-throughput screening assay (Table 4). This convergence provides mutual validation for both the screening assay results and the network pharmacology predictions.

Prioritizing among the targets identified by network pharmacology analysis, we focused on HSP90, which plays a critical regulatory role in both inflammatory processes and tumorigenesis [23,24,25,26]. Molecular docking of compound (3*S*)-**P6** with the HSP90α protein revealed that (3*S*)-**P6** fits well into the ligand-binding pocket of HSP90α (Figure 5B). The amino group at its C-3 position forms crucial hydrogen bonds with residues Asp93 and Thr184, explaining why introducing a bulky group at this site leads to a loss of anti-inflammatory activity (comparing (3*S*)-**P6** to (3*S*)-**P7**, Table 3). Furthermore, an important C-H-π interaction was observed between the angular methyl group at C-19 of the steroidal core and Phe138. Additionally, the double bond in the C-17 side chain contributed positively to enhancing the compound’s affinity for the protein. Collectively, it is presumed that compound (3*S*)-**P6** may interact with HSP90α. By interfering with the chaperone activity of HSP90α, compound (3*S*)-**P6** compromises the folding and stability of its downstream client transcription factors (STAT3, NF-κB, and HIF-1α), which ultimately contributes to its anti-inflammatory action.

## 3. Materials and Methods

### 3.1. Materials and General Synthetic Procedures

All reagents and solvents were purchased from commercial suppliers and employed without further purification. Reactions were conducted under a nitrogen atmosphere and monitored by thin-layer chromatography (TLC) on silica gel GF254 plates (Yantai Jiangyou Silica Gel Development Co., Ltd., Yantai, Shandong, China). Products were routinely purified by silica gel column chromatography (Qingdao Marine Chemical Factory, Qingdao, Shandong, China) unless otherwise noted. NMR spectra were recorded on BRUKER AVANCE NEO (Bruker BioSpin, Ettlingen, Germany) with 400 MHz or 600 MHz for proton (^1^H NMR), and 100 MHz or 150 MHz for carbon (^13^C NMR). Chemical shifts (*δ*) are reported in parts per million (ppm) relative to TMS as an internal standard; coupling constants (*J*) are given in hertz (Hz). Splitting patterns are abbreviated as s (singlet), br s (broad singlet), d (doublet), t (triplet), q (quartet), dd (doublet of doublet), and m (multiplet). High-resolution mass spectra (HRMS) were acquired on a Waters Micromass Q-TOF Premier mass spectrometer (Waters, Milford, MA, USA), and LRMS were measured with a Waters ACQUITY SQD2 Detector (Waters, Milford, MA, USA). Single-crystal X-ray diffraction data were collected using a Bruker D8 VENTURE diffractometer equipped with Cu Kα (λ = 1.54178 Å) radiation. (Bruker, Karlsruhe, Germany). ECD spectra were measured on a JASCO J-815 circular dichroism spectrometer (JASCO Corporation, Tokyo, Japan). The high-performance liquid chromatography (HPLC) analysis was conducted on an Agilent 1260 Infinity II Prime LC system (Agilent, Santa Clara, CA, USA), which was carried out on a 5-AmyCoat (4.6 × 250 mm) (Kromasil, Bohus, Sweden) with an isocratic mobile phase of 40% isopropanol in *n*-hexane at a flow rate of 1.0 mL/min. Detection was carried out by UV at 287 nm. Melting ––points of compounds were measured using an MP4 series video melting point apparatus (Hanon Advanced Technology Group Co., Ltd., Jinan, Shandong, China).

#### 3.1.1. Synthesis of (22*E*,24*R*)-Ergosta-4,7,22-trien-3-one (**P2**)

To a solution of ergosterol (**P1**) (1.19 g, 3 mmol) in dry toluene (30 mL) was added cyclohexanone (1.47 g, 15 mmol), and the reaction was stirred at room temperature for 2 h. Then the aluminium isopropoxide (0.62 g, 3 mmol) was added rapidly to the solution, and the reaction was refluxed for 1 h under nitrogen atmosphere. After completion (TLC), the solution was cooled to room temperature. The solution was quenched with addition of 2 N HCl (30 mL) at 0 °C. Then the solution was extracted with ethyl acetate (20 mL × 3). The combined organic phases were washed with water, saturated aqueous sodium bicarbonate solution, saturated aqueous sodium chloride solution, dried over anhydrous sodium sulfate, filtered and concentrated under reduced pressure. The crude residue was purified by silica gel chromatography to give the product as a white solid, mp. 98–100 °C. Yield: 0.83 g, 2.1 mmol, 70%. Compound **P2** is a known derivative, and our NMR data are in good agreement with those reported in the literature [13,16]. ^1^H NMR (400 MHz, Chloroform-*d*) *δ* 5.78 (s, 1H), 5.25–5.12 (m, 3H), 3.18–3.07 (m, 1H), 2.–2.58 (m, 1H), 2.38–2.28 (m, 2H), 2.20 (t, *J* = 8.2 Hz, 1H), 0–2.01 (m, 2H), 1–1.90 (m, 2H), 9–1.80 (m, 3H), 1.73 (d, *J* = 3.7 Hz, 1H), 1.63 (dt, *J* = 10.3, 5.1 Hz, 2H), 1.54–1.40 (m, 3H), 1.31–1.26 (m, 2H), 1.16 (s, 3H), 1.02 (d, *J* = 6.6 Hz, 3H), 0.90 (d, *J* = 6.8 Hz, 3H), 0.82 (d, *J* = 6.4 Hz, 3H), 0.80 (s, 3H), 0.59 (s, 3H). ^13^C NMR (100 MHz, Chloroform-*d*) *δ* 199.5, 169.2, 139.6, 135.5, 132.2, 122.8, 115.7, 55.9, 55.1, 45.9, 43.5, 42.9, 40.6, 39.1, 38.2, 34.3, 33.2, 33.2, 33.0, 28.2, 23.0, 22.1, 21.4, 21.2, 20.1, 19.8, 17.7, 12.3.

#### 3.1.2. Synthesis of (22*E*,24*R*)-Ergosta-4,6,8(14),22-tetraen-3-one (**P3**)

To a solution of **P2** (1.58 g, 4 mmol) in dry 1,4-dioxane (20 mL) was added DDQ (3.63 g, 16 mmol). The reaction was stirred at 110 °C for 4 h under nitrogen atmosphere. After cooling to room temperature, the mixture was filtered and the filtrate was concentrated under reduced pressure. The crude residue was purified by silica gel chromatography to give the product as a white solid, mp. 99–100 °C. Yield: 0.62 g, 1.56 mmol, 39%. Compound **P3** is a known derivative, and our NMR data are in good agreement with those reported in the literature [27]. ^1^H NMR (400 MHz, Chloroform-*d*) *δ* 6.61 (d, *J* = 9.5 Hz, 1H), 6.03 (d, *J* = 9.5 Hz, 1H), 5.73 (s, 1H), 5.31–5.17 (m, 2H), 2.60–2.33 (m, 4H), 2.18–1.99 (m, 4H), 1.91–1.77 (m, 3H), 1.75–1.61 (m, 2H), 1.53–1.43 (m, 2H), 1.31 (dd, *J* = 12.8, 2.9 Hz, 2H), 1.06 (d, *J* = 6.7 Hz, 3H), 1.00 (s, 3H), 0.96 (s, 3H), 0.93 (d, *J* = 6.8 Hz, 3H), 0.85 (t, *J* = 6.4 Hz, 3H), 0.82 (s, 3H). ^13^C NMR (100 MHz, Chloroform-*d*) *δ* 199.6, 164.5, 156.2, 135.1, 134.1, 132.7, 124.6, 124.6, 123.1, 55.9, 44.5, 44.1, 43.0, 39.4, 36.9, 35.7, 34.3, 34.3, 33.2, 27.9, 25.5, 21.4, 20.1, 19.8, 19.1, 19.1, 17.8, 16.8.

#### 3.1.3. Synthesis of (3*S*,22*E*,24*R*)-3-[(*S*)-*tert*-Butylsulfinamido]ergosta-4,6,8(14),22-tetraene (**P5a**)

To a solution of **P3** (0.98 g, 2.5 mmol) and (*S*)-(−)-2-methyl-2-propanesulfinamide (1.82 g, 15 mmol) in tetrahydrofuran (20 mL) was added titanium tetraisopropanolate (5.68 g, 20 mmol) and the reaction was refluxed for 24 h. DIBAL-H (10 mmol, 1 M in THF) was then added drop wise to the solution under nitrogen at −78 °C. After completion of the addition, the mixture was stirred at room temperature for 5 h. After the reaction was completed (detected by TLC), saturated aqueous solution of ammonium chloride was then added slowly to the reaction mixture at −78 °C and stirred for 5 min. The aqueous layer was washed with ethyl acetate (30 mL × 3). The combined organic layers were dried over anhydrous sodium sulfate, filtered and concentrated under reduced pressure. The crude residue was purified by silica gel chromatography to give the product as a pale yellow solid, mp. 142–144 °C. Yield: 0.65 g, 1.3 mmol, 52%. ^1^H NMR (400 MHz, Chloroform-*d*) *δ* 6.17 (d, *J* = 9.6 Hz, 1H), 5.87 (d, *J* = 9.7 Hz, 1H), 5.46 (s, 1H), 5.26–5.13 (m, 2H), 3.99–3.84 (m, 1H), 3.21 (d, *J* = 6.9 Hz, 1H), 2–2.35 (m, 1H), 2.34–2.24 (m, 1H), 2.16–2.09 (m, 1H), 2.09–1.94 (m, 3H), 1.90–1.72 (m, 3H), 1.67–1.5–m, 3H), 1.47–1.37 (m, 3H), 1.26 (s, 2H), 1.22 (s, 9H), 1.04 (d, *J* = 6.7 Hz, 3H), 0.97–0.90 (m, 6H), 0.89 (s, 3H), 0.86 –0.80 (m, 6H). ^13^C NMR (100 MHz, Chloroform-*d*) *δ* 150.0, 145.8, 135.4, 132.3, 126.2, 125.7, 124.9, 124.9, 56.0, 55.7, 54.2, 45.5, 43.7, 43.0, 39.6, 36.5, 35.7, 34.1, 33.2, 28.6, 28.0, 25.1, 22.8 (3C), 21.3, 20.1, 19.8, 19.3, 19.3, 18.5, 17.8. LRMS (ESI): *m*/*z* 497.980 [M + H]^+^.

#### 3.1.4. Synthesis of (3*S*,22*E*,24*R*)-3-[(*R*)-*tert*-Butylsulfinamido]ergosta-4,6,8(14),22-tetraene (**P5b**)

**P5b** was synthesized following the same procedure of **P5a** using (*R*)-(−)-2-methyl-2-propanesulfinamide as a pale-yellow solid, mp. 145–148 °C. Yield: 0.77 g, 1.55 mmol, 62%. ^1^H NMR (400 MHz, Chloroform-*d*) *δ* 6.17 (d, *J* = 9.6 Hz, 1H), 5.85 (d, *J* = 9.7 Hz, 1H), 5.33 (s, 1H), 5.27–5.15 (m, 2H), 3.97–3.84 (m, 1H), 3.09 (d, *J* = 9.1 Hz, 1H), 2.46–2.35 (m, 1H), 2.35–2.23 (m, 2H), 2.17–2.06 (m, 1H), 2.05–1.92 (m, 2H), 1.86 (td, *J* = 6.9, 5.8 Hz, 1H), 1.81–1.71 (m, 2H), 1.60 (ddt, *J* = 16.0, 9.2, 3.1 Hz, 2H), 1.54–1.37 (m, 4H), 1.30–1.25 (m, 1H), 1.22 (s, 9H), 1.20–1.16 (m, 1H), 1.04 (d, *J* = 6.7 Hz, 3H), 0.95–0.89 (m, 6H), 0.87 (s, 3H), 0.85–0.80 (m, 6H). ^13^C NMR (100 MHz, Chloroform-*d*) *δ* 150.2, 145.9, 135.5, 132.3, 126.3, 125.6, 124.9, 124.6, 56.0, 55.9, 55.0, 45.5, 43.7, 43.0, 39.5, 36.5, 35.6, 34.3, 33.2, 29.9, 28.0, 25.2, 22.8 (3C), 21.4, 20.1, 19.8, 19.3, 19.3, 18.6, 17.8. LRMS (ESI): *m*/*z* 498.520 [M + H]^+^.

#### 3.1.5. Synthesis of (3*S*,22*E*,24*R*)-Ergosta-4,6,8(14),22-tetraen-3-amine hydrochloride ((3*S*)-**P6**)

To a solution of **P5a** or **P5b** (200 mg, 0.4 mmol) in 1,4-dioxane (2 mL) was added 2 M HCl in 1,4-dioxane (3 mL). The resulting mixture was stirred at room temperature for 4 h. The solution was concentrated and then diluted with MeOH (10 mL) and filtrated. The solid was washed with petroleum ether and then dried overnight to give (3*S*)-**P6** as a white solid, mp. 112–114 °C. Yield: 149 mg, 0.32 mmol, 80%. ^1^H NMR (400 MHz, Chloroform-*d*) *δ* 8.41 (br s, 2H), 6.21 (d, *J* = 9.5 Hz, 1H), 5.88 (d, *J* = 9.7 Hz, 1H), 5.50 (s, 1H), 5.29–5.14 (m, 2H), 4.13–3.87 (m, 1H), 2.47–2.36 (m, 1H), 2.34–2.21 (m, 2H), 2.16–2.08 (m, 1H), 2.06–1.90 (m, 3H), 1.89–1.83 (m, 1H), 1.80–1.74 (m, 2H), 1.60–1.56 (m, 1H), 1.54–1.34 (m, 4H), 1.25–1.15 (m, 2H), 1.04 (d, *J* = 6.6 Hz, 3H), 0.95–0.88 (m, 9H), 0.86–0.80 (m, 6H). ^13^C NMR (100 MHz, Chloroform-*d*) *δ* 150.9, 148.6, 135.4, 132.4, 127.3, 125.3, 124.6, 117.8, 77.4, 56.0, 49.4, 45.1, 43.8, 43.1, 39.6, 36.4, 35.8, 33.4, 33.3, 28.0, 25.2, 21.4, 20.1, 19.8, 19.3, 19.3, 18.5, 17.8. LRMS (ESI): *m*/*z* 394.37 [M + H]^+^.

#### 3.1.6. Synthesis of *N*4-[(3*S*,22*E*,24*R*)-Ergosta-4,6,8(14),22-tetraen-3-yl]-6-chloropyrimidine-4,5-diamine ((3*S*)-**P7**)

A solution of (3*S*)-**P6** (93 mg, 0.2 mmol) and 5-amino-4,6-dichloropyrimidine (36 mg, 0.22 mmol) in *n*-butanol (5 mL) was treated with sodium bicarbonate (50 mg, 0.6 mmol) and refluxed for 24 h. After the reaction was completed, the solution was quenched with H_2_O. Then the solution was extracted with ethyl acetate (15 mL × 3). The organic layers were combined and washed with water, saturated aqueous sodium chloride solution, dried over anhydrous sodium sulfate, filtered and concentrated under reduced pressure. The crude residue was purified by silica gel chromatography to give the product as a pale yellow solid, mp. 128–130 °C. Yield: 68 mg, 0.13 mmol, 66%. ^1^H NMR (400 MHz, Chloroform-*d*) *δ* 8.07 (s, 1H), 6.18 (d, *J* = 9.6 Hz, 1H), 5.84 (d, *J* = 9.7 Hz, 1H), 5.41–5.34 (m, 1H), 5.30–5.15 (m, 2H), 4.84 (br s, 2H), 3.48–3.27 (m, 2H), 2.48–2.36 (m, 1H), 2.35–2.22 (m, 2H), 2.16–2.08 (m, 1H), 2.07–1.97 (m, 2H), 1.90–1.84 (m, 1H), 1.77 (tt, *J* = 9.5, 3.5 Hz, 2H), 1.63 (td, *J* = 6.4, 2.9 Hz, 1H), 1.57–1.41 (m, 5H), 1.34–1.28 (m, 1H), 1.23–1.18 (m, 1H), 1.05 (d, *J* = 6.7 Hz, 3H), 0.97–0.88 (m, 9H), 0.86–0.81 (m, 6H). ^13^C NMR (100 MHz, Chloroform-*d*) *δ* 154.9, 150.2, 150.0, 146.3, 143.4, 135.4, 132.4, 126.3, 125.6, 124.9, 123.1, 121.7, 56.0, 48.8, 45.5, 43.7, 43.0, 39.5, 36.5, 35.8, 34.1, 33.2, 28.0, 26.7, 25.2, 21.4, 20.1, 19.8, 19.4, 19.3, 18.6, 17.8. LRMS (ESI): *m*/*z* 521.32 [M + H]^+^.

#### 3.1.7. Synthesis of 6-Chloro-9-[(3*S*,22*E*,24*R*)-ergosta-4,6,8(14),22-tetraen-3-yl]-9*H*-purine ((3*S*)-**P8**)

Conc. HCl (30 μL) was added to a solution of (3*S*)-**P7** (52 mg, 0.1 mmol) in trimethoxymethane (2 mL). The reaction was stirred for 6 h at room temperature and then concentrated *in vacuo*. The crude residue was purified by silica gel chromatography to give the product as a white solid, mp. 166–167 °C. Yield: 34 mg, 0.064 mmol, 64%. ^1^H NMR (400 MHz, Chloroform-*d*) *δ* 8.74 (s, 1H), 8.19 (s, 1H), 6.31 (d, *J* = 9.6 Hz, 1H), 5.92 (d, *J* = 9.7 Hz, 1H), 5.49–5.43 (m, 1H), 5.42–5.39 (m, 1H), 5.32–5.16 (m, 2H), 2.49–2.32 (m, 3H), 2.18–2.03 (m, 3H), 1.97–1.75 (m, 4H), 1.72–1.68 (m, 1H), 1.64–1.58 (m, 2H), 1.56–1.42 (m, 3H), 1.24–1.19 (m, 1H), 1.06 (d, *J* = 6.6 Hz, 3H), 1.03 (s, 3H), 0.96 (s, 3H), 0.93 (d, *J* = 6.8 Hz, 3H), 0.86–0.82 (m, 6H). ^13^C NMR (100 MHz, Chloroform-*d*) *δ* 151.9, 151.8, 151.1, 149.1, 146.1, 143.7, 135.3, 132.5, 132.0, 127.9, 124.6, 124.4, 118.7, 56.0, 53.0, 45.3, 43.8, 43.0, 39.5, 36.3, 35.9, 33.9, 33.2, 28.0, 27.6, 25.3, 21.4, 20.1, 19.8, 19.3, 19.3, 18.7, 17.8. LRMS (ESI): *m*/*z* 531.39 [M + H]^+^.

#### 3.1.8. Synthesis of 9-[(3*S*,22*E*,24*R*)-Ergosta-4,6,8(14),22-tetraen-3-yl]-9*H*-purin-6-amine ((3*S*)-peniadenisteroid A)

To a solution of (3*S*)-**P8** (27 mg, 0.05 mmol) in THF (1 mL) was added ammonia (7 M in MeOH) and saturated with ammonia in a sealed tube. The resulting solution was then heated to 70 °C and stirred for 30 h. Upon reaching room temperature, the reaction mixture was unsealed, monitored for completion, and then concentrated under reduced pressure. The residue was purified by silica gel chromatography to give the product as a white solid, mp. 238–240 °C. Yield: 18 mg, 0.035 mmol, 69%. NMR data were shown in Table 2. HRMS (ESI) for C_33_H_46_N_5_ [M + H]^+^ calcd 512.3748, found 512.3751.

### 3.2. General Procedures of Biological Studies

#### 3.2.1. Nitric Oxide (NO) Production Inhibitory Activity

Nitric oxide (NO) production inhibitory activity was evaluated using the Griess assay as previously described [28]. Briefly, RAW264.7 cells (kindly provided by Prof. Chang-Lun Shao, Ocean University of China) were seeded into 96-well plates at a density of 1.5 × 10^5^ cells/mL (100 μL per well) and incubated overnight at 37 °C in a 5% CO_2_ atmosphere. The cells were then treated with test compounds or dexamethasone (Macklin, Shanghai, China, positive control) at 10 μM in DMEM (Biosharp, Beijing, China) supplemented with 10% FBS (Biological Industries, Kibbutz Beit Haemek, Israel) for 24 h. Subsequently, the cells were stimulated with 1 μg/mL lipopolysaccharide (LPS, from *E. coli*, Promega (Beijing), Beijing, China) to induce NO production. The concentration of nitrite in the culture supernatant, reflecting NO levels, was determined colorimetrically based on the Griess reaction. In each experiment, NO release in the LPS-stimulated group was defined as 100%, and that in the unstimulated group was defined as 0%. All experiments were performed in triplicate and repeated three times independently.

#### 3.2.2. Pathway Reporter Assay

SKA cells [29] were plated in white 96-well plates at a density of 10^4^ cells per well and cultured overnight under standard conditions (37 °C, 5% CO_2_). Following attachment, the cells were incubated for 24 h with the test compounds: (3*S*)-**P6** (at 20 μM) and KC7F2 (Selleck (Shanghai, China), at 20 μM), and Liangshanin A [30] (at 7.5 μM). Subsequently, luciferase activity was quantified using a commercial detection kit (Promega (Beijing), Beijing, China, product #E1483) and measured on a SpectraMax® L microplate reader (Molecular Devices, San Jose, CA, USA).

### 3.3. Molecular Docking Study

Molecular docking simulations were conducted in Molecular Operating Environment (MOE, version 2020.09) to explore the potential binding interactions between compound (3*S*)-**P6** and its target proteins. The three-dimensional structure of each protein in complex with a reference ligand was obtained from the RCSB Protein Data Bank. Prior to docking, all water molecules and ions were deleted from the protein structure. Protein preparation was carried out using the QuickPrep module, which assigned protonation states, optimized hydrogen-bonding networks, and performed energy minimization. The binding site was defined based on the coordinates of the co-crystallized ligand. During the docking procedure, poses were initially generated and ranked using the London dG scoring function, then refined and rescored with the more accurate GBVI/WSA dG scoring method. The conformation exhibiting the most favorable binding free energy was selected as the predicted binding mode. The resulting PDB file from docking was imported into PyMOL (version 2.6.0) for processing. Key intermolecular interactions were visualized using the “find contacts” function, which identifies contacts between the ligand and the protein.

## 4. Conclusions

In this study, we accomplished the semi-synthesis of (3*S*)-peniadenisteroid A-the C-3 epimer of the natural product peniadenisteroid A-from ergosterol via an eight -step sequence in 3.9% overall yield. Although the total synthesis of the natural 3*R* product remains incomplete, this work provides a foundational step and a valuable reference for its eventual realization. Among the synthesized compounds, (3*S*)-**P6** exhibited excellent inhibitory activity against NO production (86.1% at 10 μM), markedly outperforming the positive control dexamethasone (35.1% at 10 μM). Further high-throughput signaling pathway screening revealed that (3*S*)-**P6** significantly suppressed both the STAT3/NF-κB and HIF-1α pathways. Integrated network pharmacology analysis of 141 intersecting targets identified 10 highly correlated candidates. Ultimately, by integrating network pharmacology and molecular docking, we postulate that HSP90α may serve as a putative target of compound (3*S*)-**P6**. We propose that the anti-inflammatory mechanism of (3*S*)-**P6** may involve inhibition of HSP90α, which disrupts the chaperone-mediated maturation of key client transcription factors (STAT3, NF-κB, and HIF-1α), resulting in a synergistic multi-pathway effect [23,24,25,26].

In summary, our exploration of the synthetic routes toward the natural product and its diastereomer offers key guidance for the synthesis and structural diversification of this class of compounds. These findings provide a solid chemical and mechanistic foundation for the development of novel small-molecule anti-inflammatory agents based on marine natural product structures, and point the way toward designing multi-pathway modulators targeting the STAT3/NF-κB, HIF-1α network.

## Figures and Tables

**Table 1:** Optimization of reaction conditions for the synthesis of compound **P3**.

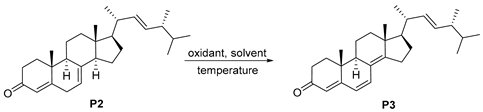						
**Entry**	**Reagent**	**Equivalent**	**Temperature**	**Time**	**Solvent**	**Yield**
1	Jones	4	25 °C	12 h	THF	trace
2	Jones	4	40 °C	12 h	Toluene	7%
3	Jones	4	40 °C	12 h	1,4-dioxane	11%
4	Jones	8	40 °C	12 h	1,4-dioxane	8%
5	PCC	4	25 °C	12 h	CH_2_Cl_2_	8%
6	PCC	4	40 °C	12 h	1,4-dioxane	trace
7	PCC	8	40 °C	12 h	1,4-dioxane	trace
8	PDC	4	25 °C	12 h	CH_2_Cl_2_	trace
9	IBX	4	25 °C	12 h	CH_2_Cl_2_	trace
10	IBX	4	40 °C	12 h	1,4-dioxane	trace
11	DMP	4	25 °C	12 h	CH_2_Cl_2_	trace
12	DMP	4	40 °C	12 h	1,4-dioxane	trace
13	DDQ	4	25 °C	12 h	CH_2_Cl_2_	6%
14	DDQ	4	25 °C	36 h	CH_2_Cl_2_	10%
15	DDQ	4	40 °C	12 h	1,4-dioxane	19%
16	DDQ	4	110 °C	4 h	1,4-dioxane	39%
17	DDQ	4	110 °C	6 h	1,4-dioxane	30%

**Table 2:** ^1^H NMR and ^13^C NMR data of compound peniadenisteroid A and (3*S*)-peniadenisteroid A.

Position	Striasteroid C (CDCl_3_, *δ* in ppm) ^a^		Peniadenisteroid A (CDCl_3_, *δ* in ppm) ^b^		(3*S*)-Peniadenisteroid A (CDCl_3_, *δ* in ppm) ^c^	
	^1^H NMR	^13^C NMR	^1^H NMR	^13^C NMR	^1^H NMR	^13^C NMR
1	1.62 ^d^ 1.31 ^d^	29.5	1.62 (m) 1.30 (m)	29.6	1.79 (m) 1.45 (m)	33.2
2	2.03 (m) 2.23 (m)	25.6	2.03 (m) 2.23 (m)	25.7	1.93 (m) 2.33 (m)	27.9
3	5.32 (t, 5.0)	49.1	5.31 (t, 5.1)	49.0	5.37 (t, 5.0)	52.0
4	5.56 (d, 5.0)	116.7	5.56 (d, 4.9)	116.8	5.44 (d, 4.8)	119.9
5	-	150.4	-	150.4	-	150.0
6	6.00 (d, 9.5)	124.8	6.00 (d, 9.7)	124.8	5.92 (d, 9.7)	124.9
7	6.35 (d, 9.5)	128.3	6.35 (d, 9.6)	128.3	6.28 (d, 9.6)	127.4
8	-	124.4	-	124.4	-	124.5
9	2.10 (m)	45.4	2.10 (m)	45.5	2.10 (m)	45.3
10	-	36.4	-	36.1	-	35.8
11	1.62 ^d^ 1.56 (m)	19.6	1.63 (m) 1.56 (m)	19.6	1.63 (m) 1.57 (m)	19.3
12	2.05 (m) 1.31^d^	36.1	2.04 (m) 1.33 (m)	36.4	2.04 (m) 1.31 (m)	36.3
13	-	43.9	-	43.9	-	43.8
14	-	151.9	-	151.8	-	151.3
15	2.36 (m) 2.46 (m)	25.3	2.36 (m) 2.46 (m)	25.3	2.38 (m) 2.47 (m)	25.2
16	1.81 (m) 1.48 ^d^	28.0	1.80 (m) 1.48 (m)	28.0	1.84 (m) 1.50 (m)	28.0
17	1.25 (m)	56.0	1.24 (m)	56.0	1.24 (m)	56.0
18	0.94 (s)	19.3	0.94 (s)	19.3	0.95 (s)	19.3
19	0.95 (s)	17.8	0.95 (s)	17.8	1.01 (s)	18.7
20	2.15 (m)	39.5	2.14 (m)	39.5	2.15 (m)	39.5
21	1.05 (d, 7.0)	21.4	1.05 (d, 6.7)	21.4	1.06 (d, 6.7)	21.4
22	5.21 (dd, 15.0, 8.0)	135.3	5.20 (dd, 15.3, 7.8)	135.3	5.21 (m)	135.4
23	5.26 (dd, 15.0, 7.0)	132.4	5.25 (dd, 15.2, 7.1)	132.5	5.24 (m)	132.4
24	1.88 (m)	43.0	1.87 (m)	43.0	1.88 (m)	43.0
25	0.93 (d, 7.0)	17.8	0.93 (d, 7.8)	17.8	0.93 (d, 6.8)	17.8
26	1.48 ^d^	33.2	1.46 (m)	33.2	1.68 (m)	34.0
27	0.83 (d, 7.0)	20.1	0.83 (d, 6.8)	20.1	0.83 (d, 6.6)	20.1
28	0.85 (d, 7.0)	19.8	0.85 (d, 6.7)	19.8	0.85 (d, 6.6)	19.8
29	7.79 (s)	140.3	7.78 (s)	140.3	7.88 (s)	138.8
30	-	120.2	-	120.4	-	119.9
31	-	155.5	-	155.6	-	155.6
32	8.36 (s)	152.7	8.38 (s)	152.9	8.36 (s)	152.9
33	-	149.5	-	149.6	-	148.4
NH_2_	5.83 (br s)	-	5.77 (br s)	-	5.71 (br s)	-

^a^ Data were obtained from reference [12]. ^b^ NMR spectra were recorded on a BRUKER AVANCE NEO (^1^H, 600 MHz; ^13^C, 150 MHz) spectrometer. ^c^ NMR spectra were recorded on a BRUKER AVANCE NEO (^1^H, 400 MHz; ^13^C, 100 MHz) spectrometer. ^d^ Overlapped signal [12].

**Table 3:** Anti-inflammatory activity against LPS-induced NO production in RAW264.7 macrophages of compounds ^a^.

Compounds	Inhibition Rate at 10 µM (%)
**P2**	20.5 ± 6.9
**P3**	59.7 ± 8.5
**P5a**	28.5 ± 12.4
(3*S*)-**P6**	86.1 ± 5.6
(3*S*)-**P7**	19.3 ± 11.8
(3*S*)-**P8**	21.5 ± 8.8
(3*S*)-peniadenisteroid A	39.1 ± 10.3
peniadenisteroid A	12.2 ± 3.4
Dexamethasone	35.1 ± 8.6

^a^ The values are the mean ± SEM of at least three independent experiments.

**Table 4:** Effects of (3*S*)-**P6** on STAT3/NF-kB and HIF-1α signaling pathways.

Compounds	STAT3/NF-Kb Logratio ^a^	HIF-1α Logratio ^a^
(3*S*)-**P6** ^c^	−11.74	−9.19
KC7F2 ^c^	NT ^b^	−3.09
Liangshanin A ^d^	−4.15	NT ^b^

^a^ Compared with the luciferase in the untreated control group, the fold change of luciferase in the drug-treated group was measured as log_2_FoldChange (log_2_FC). ^b^ NT means not tested. ^c^ Concentration for compound is 20 μM. ^d^ Concentration for compound is 7.5 μM.

## Data Availability

The data presented in this study are available in the manuscript and in the Supplementary Materials.

## Supplementary Materials

The following supporting information can be downloaded at: https://www.mdpi.com/article/10.3390/md24100352/s1, Figures S1–S18: ^1^H and ^13^C NMR spectra of relevant compounds; Figure S19: HRMS-ESI spectra of compound (3*S*)-Peniadenisteroid A; Figures S20 and S21: LRMS-ESI spectra of compounds **P5a** and **P5b**; Figures S22 and S23: 2D NOESY spectra of compounds **P5a** and **P5b**; Figure S24: Venn diagram of overlapping targets for (3*S*)-P6.
