# Supplementary material for: Synthesis and Biological Evaluation of a Marine Steroidal Alkaloid Analog (3S)-Peniadenisteroid A
Source: Mar Drugs. 2026 Oct 6;24(10):352. doi: 10.3390/md24100352 (PMC13706293; doi:10.3390/md24100352)
Supplement: Supplementary file 1 [file marinedrugs-24-00352-s001.zip › marinedrugs-4597006-supplementary.pdf]

Synthesis and Biological Evaluation of a Marine Steroidal Alkaloid Analog (3*S*)-Peniadenisteroid A

Xuetao Zhang[1,†], Xiangyu Wang[1,†], Mireguli Maimaitiming[1,3], Jichen Yang[1], Jie Wang[1], Danling Gao[1], Lujia Yang[1], Xinxin Zhang[4], Zhiqing Liu[1,*], Pingyuan Wang[1,*], Chang-Yun Wang[1,2,*]

[1]Key Laboratory of Marine Drugs and Key Laboratory of Evolution and Marine Biodiversity (Ministry of Education), School of Medicine and Pharmacy, Institute of Evolution & Marine Biodiversity, Ocean University of China, Qingdao 266003, China
[2]Laboratory for Marine Drugs and Bioproducts, Qingdao Marine Science and Technology Center, Qingdao 266237, China
[3]Department of Forensic Medicine, College of Basic Medical Sciences, Xinjiang Medical University, Urumqi 830011, China
[4]Marine Biomedical Research Institute of Qingdao, School of Medicine and Pharmacy, Ocean University of China, Qingdao 266003, China
*Correspondence: wangpingyuan@ouc.edu.cn (P.W.); liuzhiqing@ouc.edu.cn (Z.L.); changyun@ouc.edu.cn (C.-Y.W.)
[†]These authors contributed equally to this work and should be considered as co-first authors

Table of Contents

Figure S1 - S18. ^{1}H and ^{13}C NMR spectra of relevant compounds.. S2 - S10
Figure S19. HRMS-ESI spectra of compound (3*S*)-Peniadenisteroid A... S11
Figure S20 - S21. LRMS-ESI spectra of compounds **P5a** and **P5b**... S12
Figure S22 - S23. 2D NOESY spectra of compounds **P5a** and **P5b**... S13
Figure S24. Venn diagram of overlapping targets for (3*S*)-**P6**……………………….............................. S14

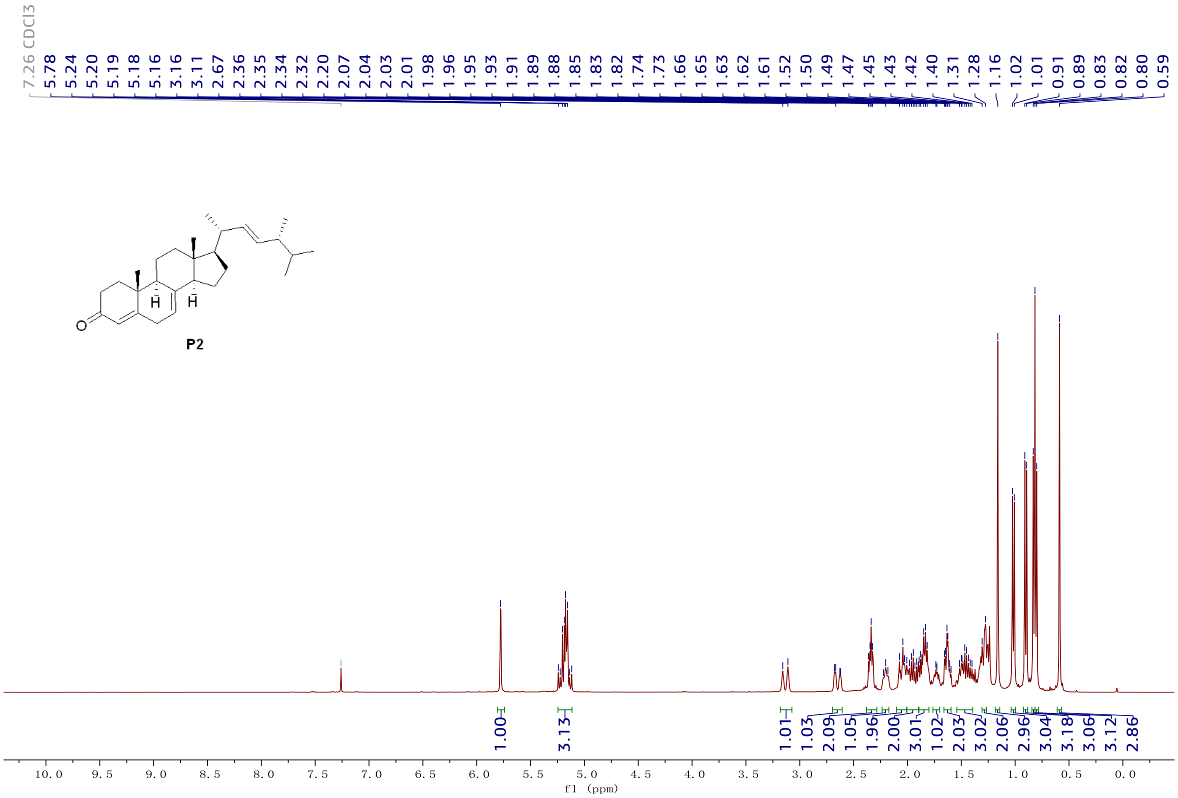

Figure S1. ^{1}H NMR spectrum (400 MHz, $CDCl_3$) of compound P2

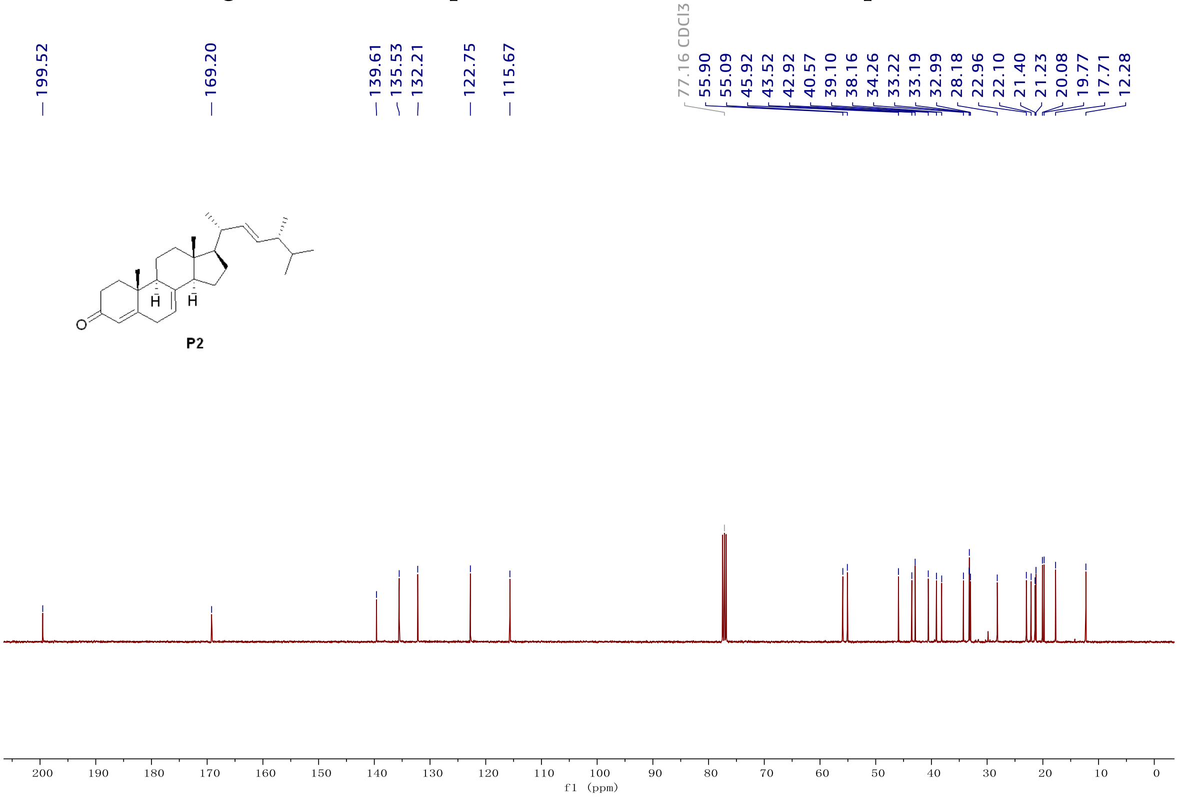

Figure S2. ^{13}C NMR spectrum (100 MHz, $CDCl_3$) of compound P2

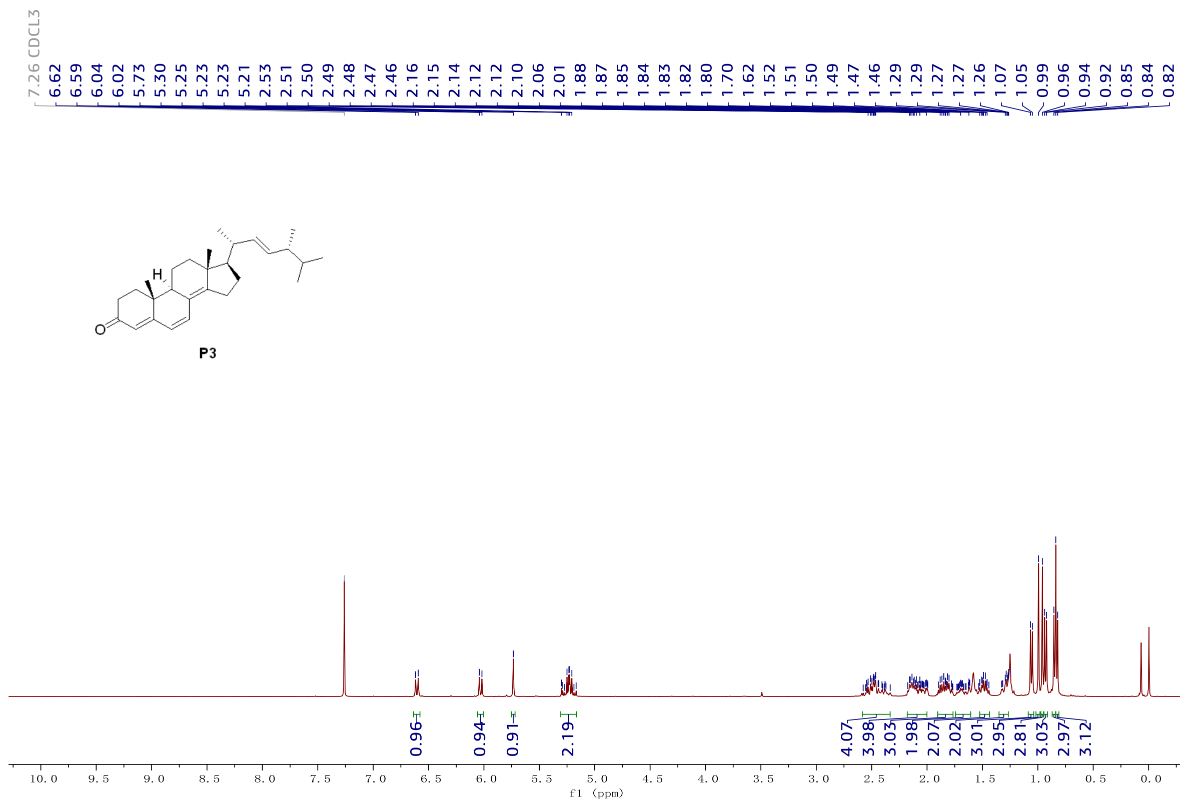

Figure S3. ^{1}H NMR spectrum (400 MHz, $CDCl_3$) of compound P3

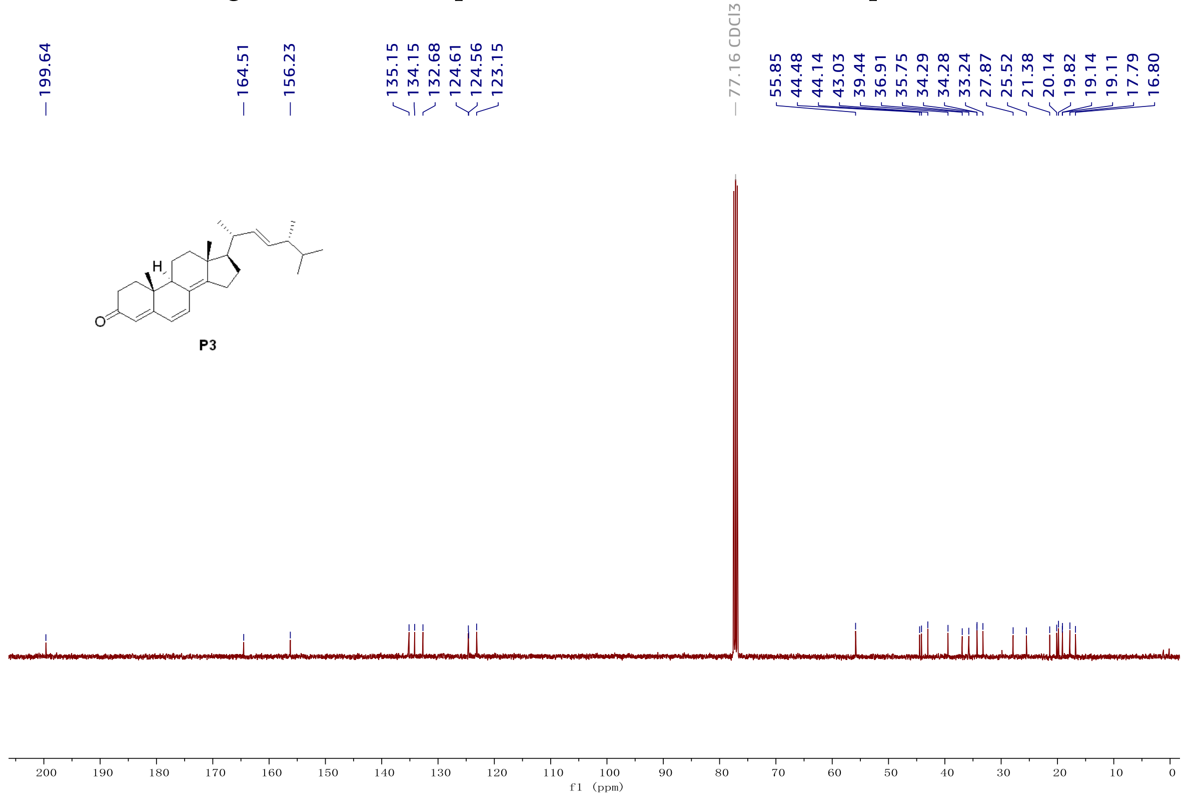

Figure S4. ^{13}C NMR spectrum (100 MHz, $CDCl_3$) of compound P3

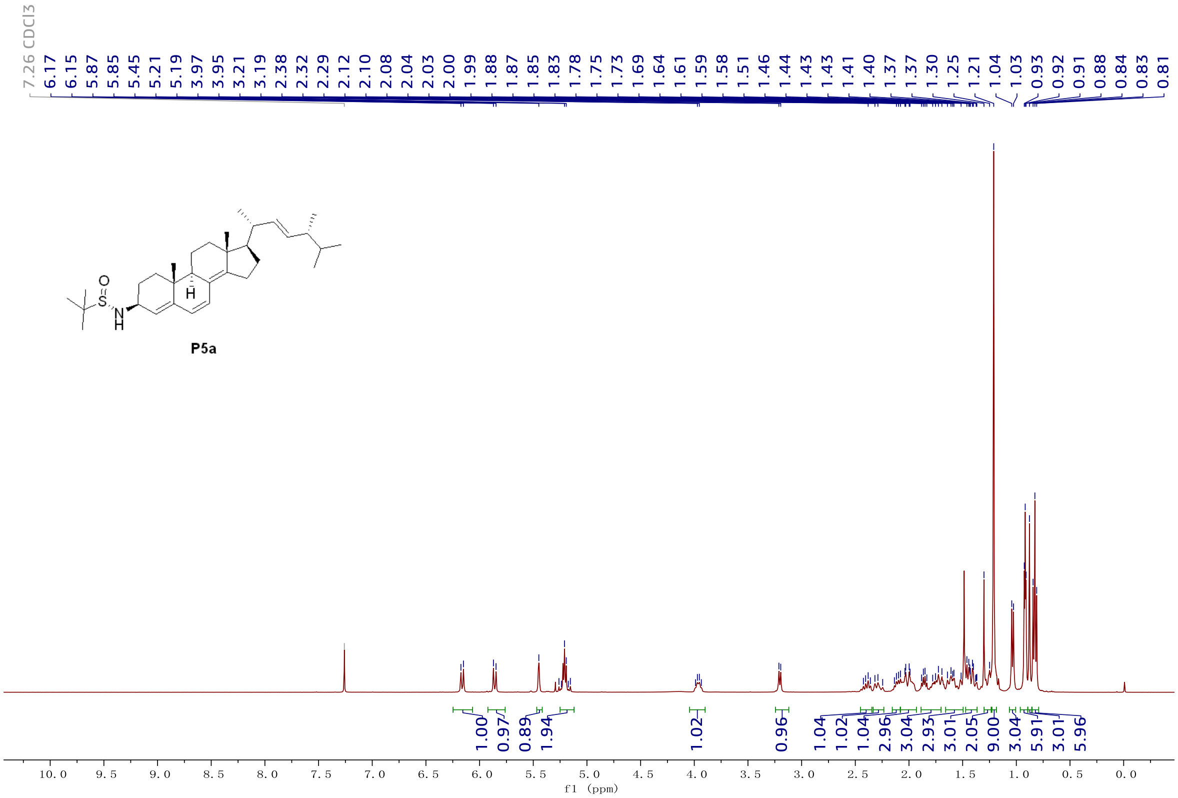

Figure S5. ^{1}H NMR spectrum (400 MHz, $CDCl_3$) of compound P5a

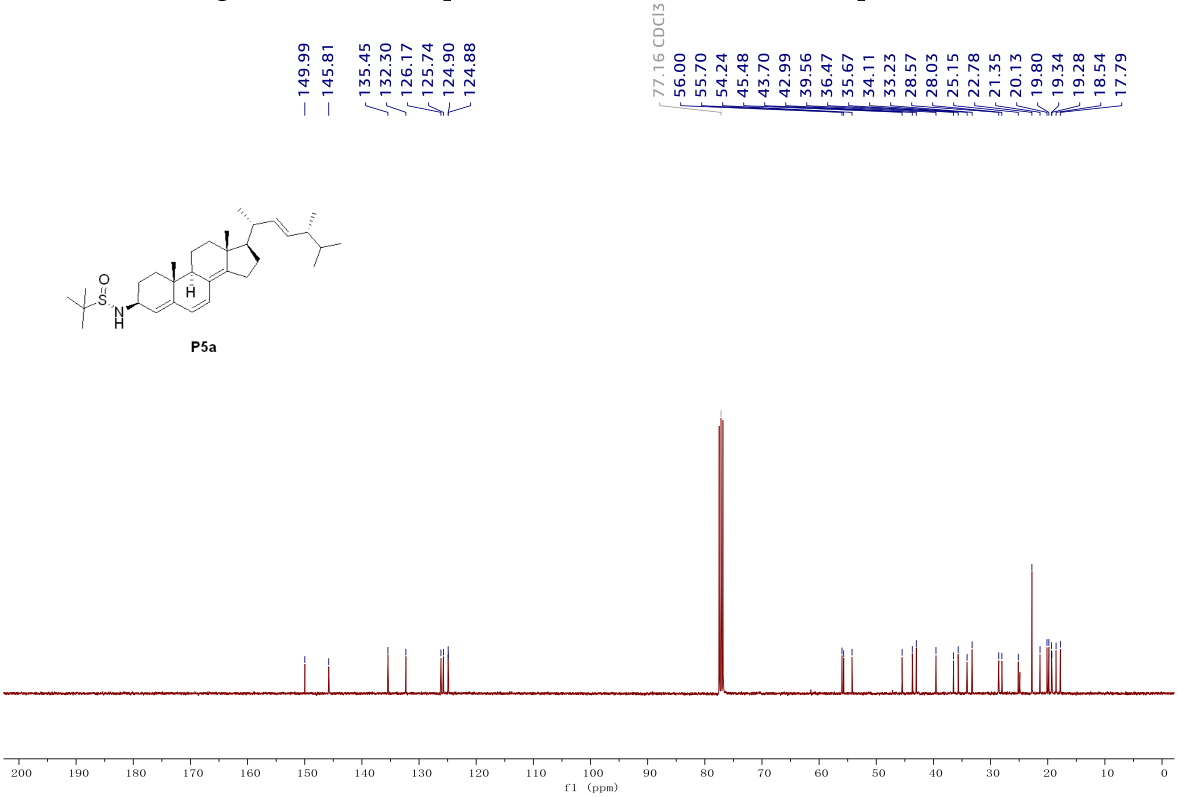

Figure S6. ^{13}C NMR spectrum (100 MHz, $CDCl_3$) of compound P5a

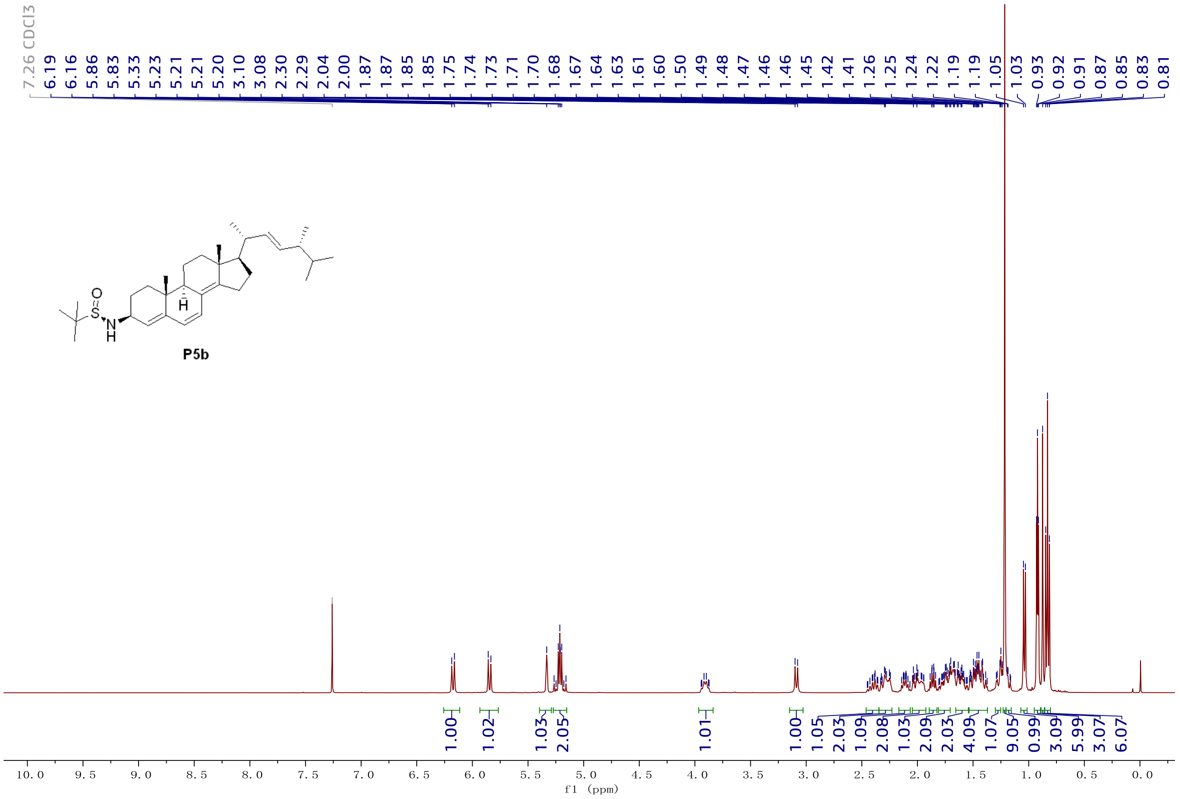

Figure S7. ^{1}H **NMR spectrum (400 MHz,** $CDCl_3$**) of compound P5b**

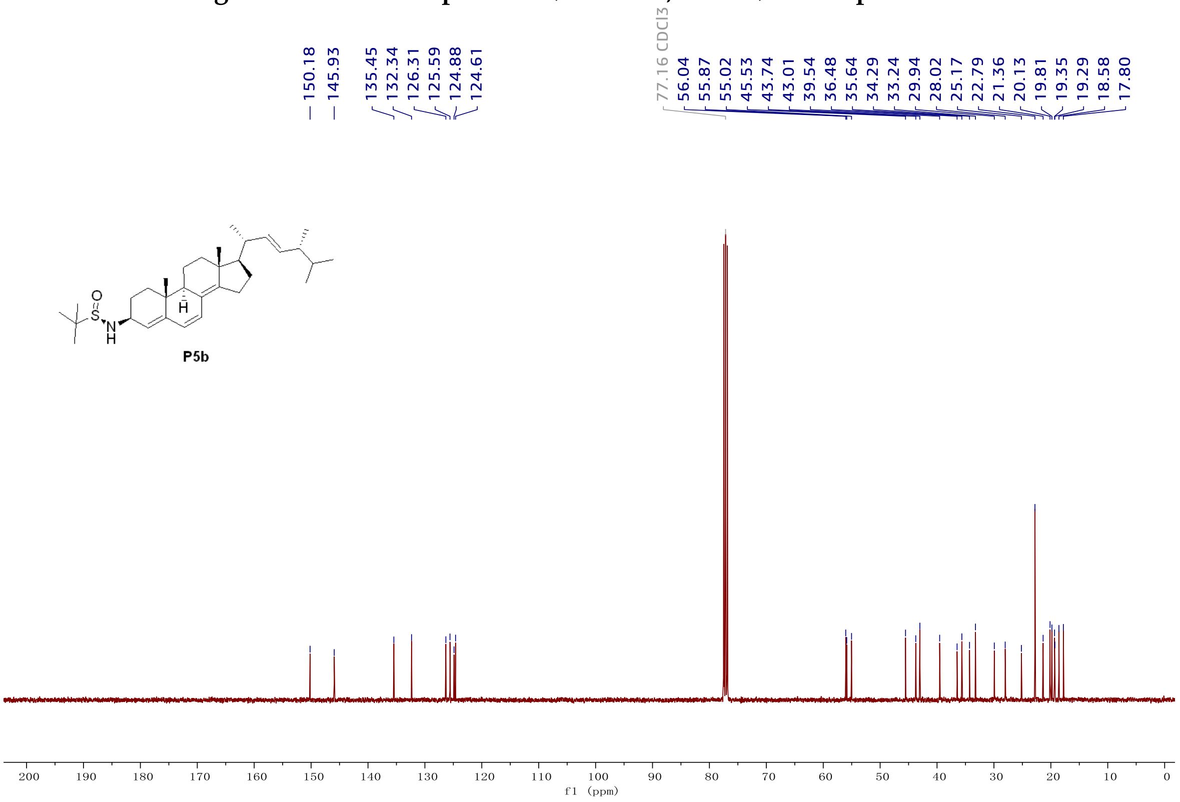

Figure S8. ^{13}C **NMR spectrum (100 MHz,** $CDCl_3$**) of compound P5b**

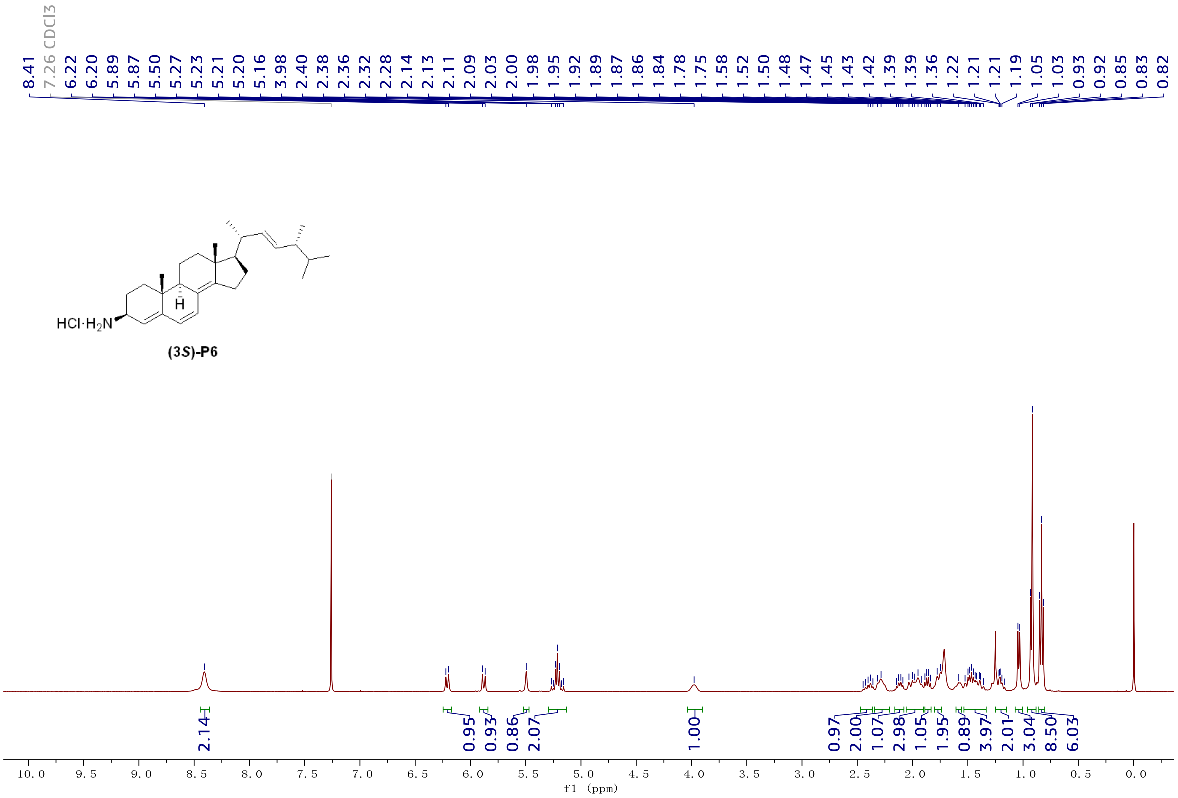

Figure S9. **^{1}H NMR spectrum (400 MHz, $CDCl_3$) of compound (3*S*)-P6**

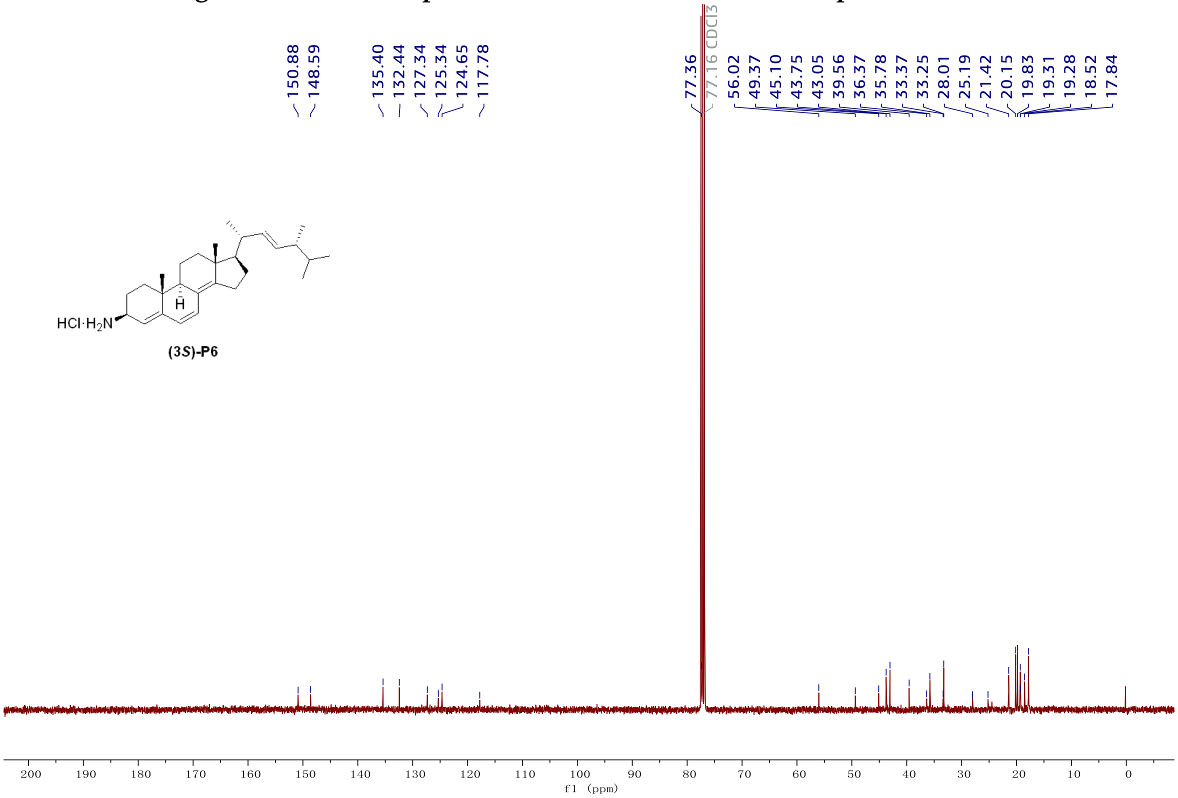

Figure S10. **^{13}C NMR spectrum (100 MHz, $CDCl_3$) of compound (3*S*)-P6**

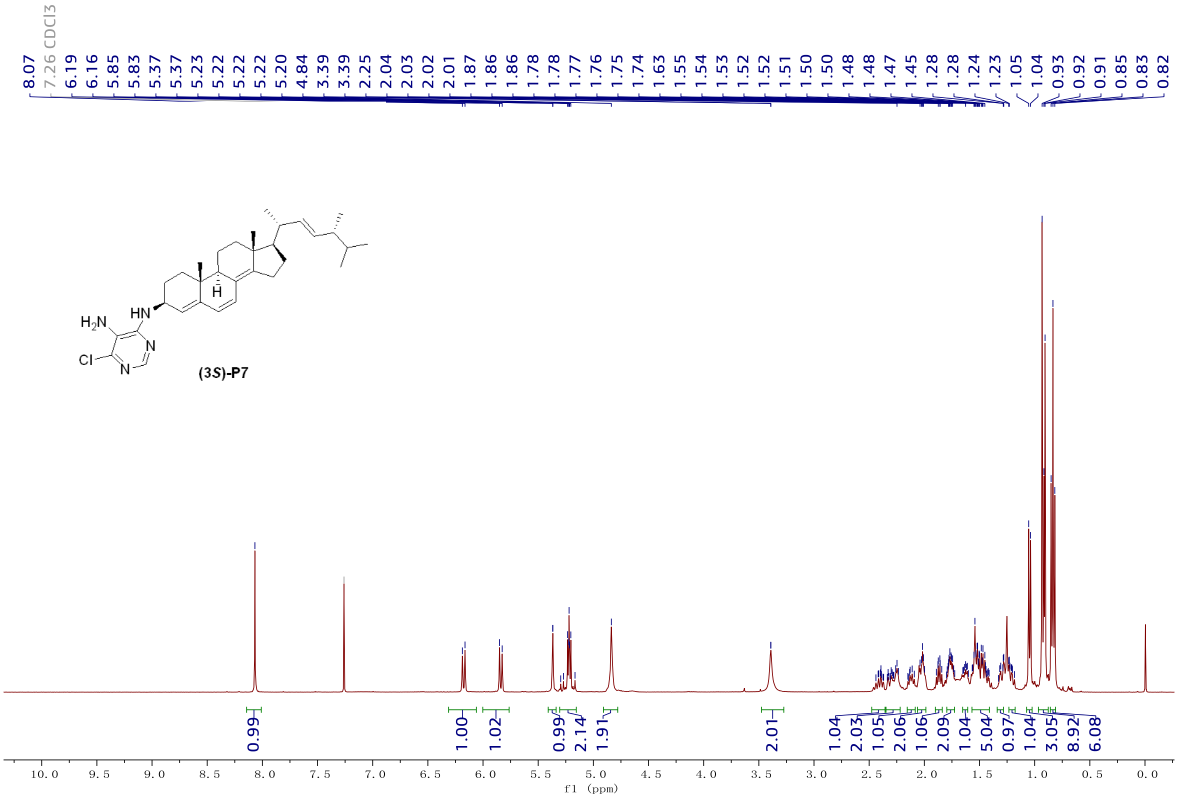

Figure S11. ^{1}H **NMR spectrum (400 MHz,** $CDCl_3$**) of compound (3*S*)-P7**

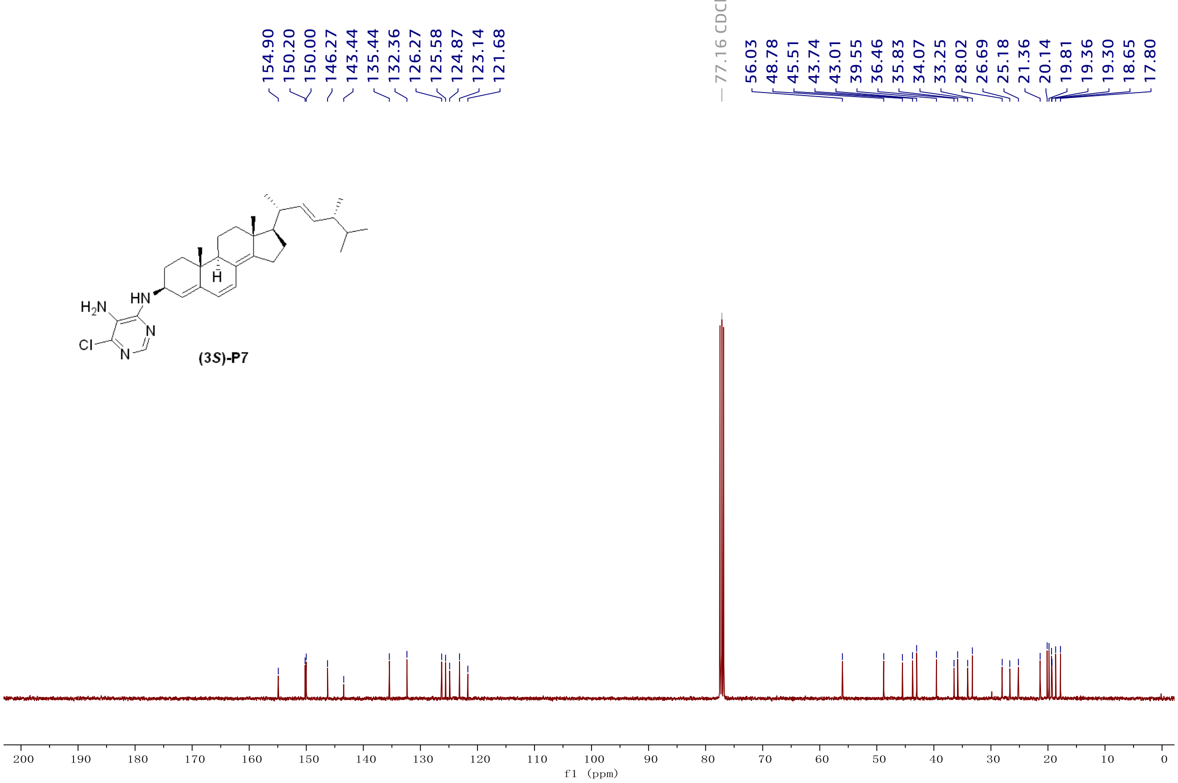

Figure S12. ^{13}C **NMR spectrum (100 MHz,** $CDCl_3$**) of compound (3*S*)-P7**

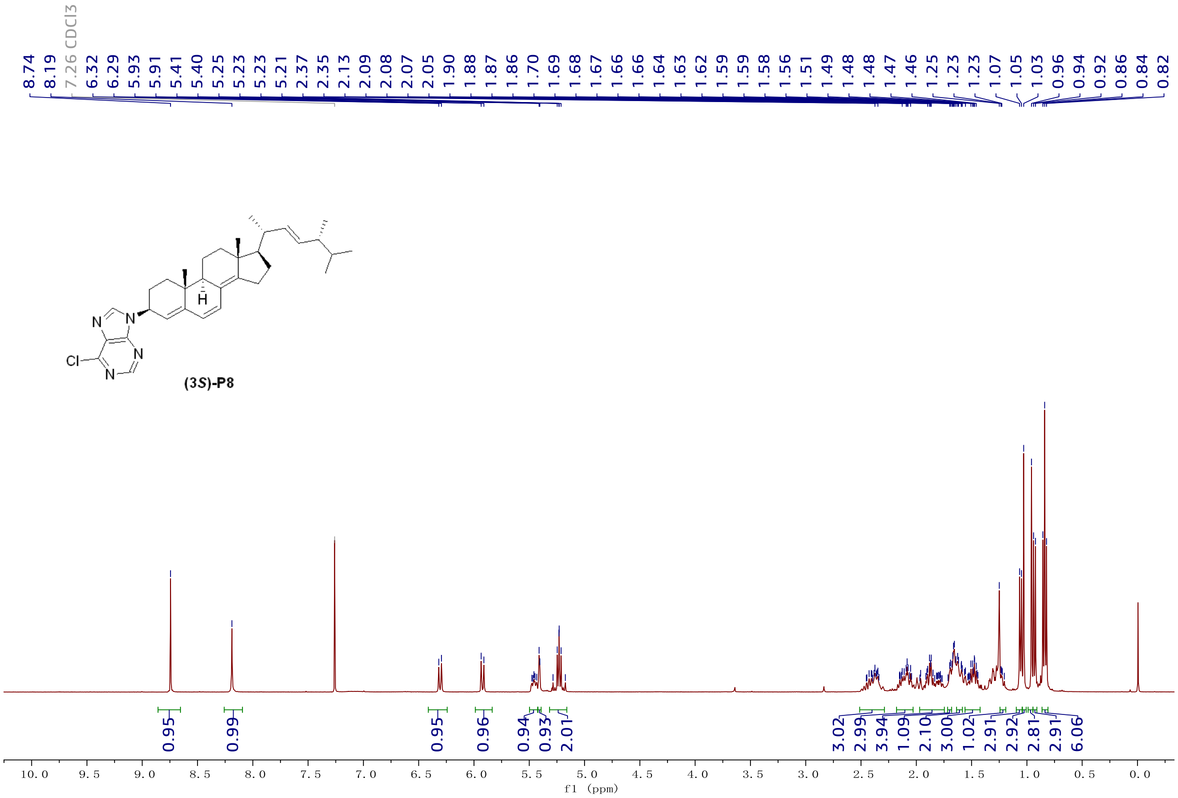

Figure S13. ^{1}H **NMR spectrum (400 MHz, $CDCl_3$) of compound (3*S*)-P8**

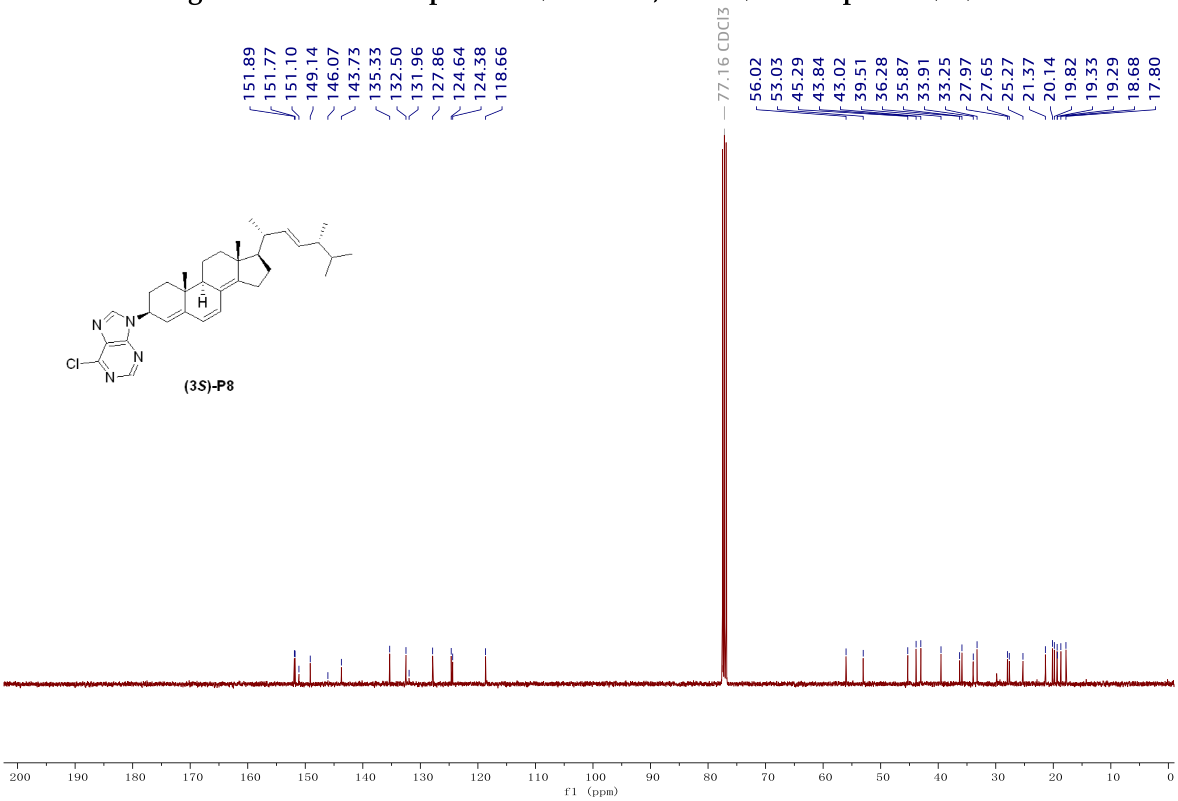

Figure S14. ^{13}C **NMR spectrum (100 MHz, $CDCl_3$) of compound (3*S*)-P8**

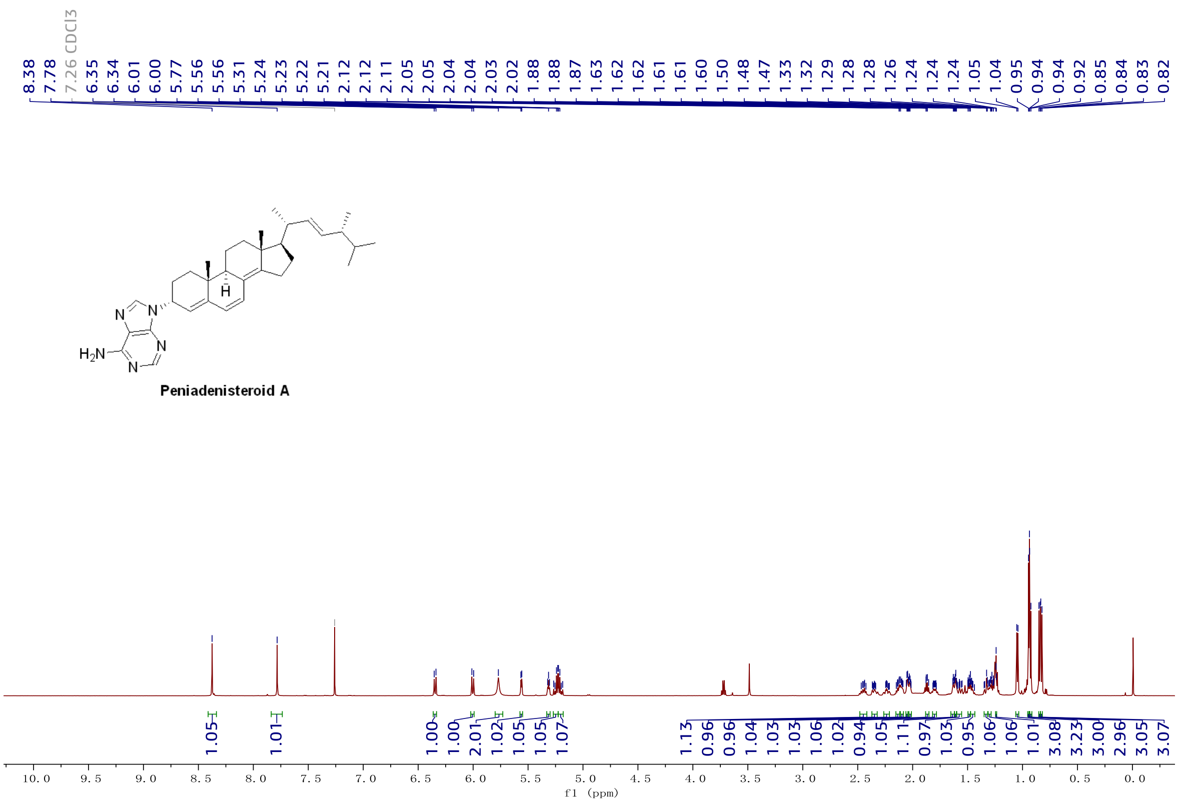

Figure S15. ^1H **NMR spectrum (600 MHz,** $CDCl_3$**) of compound Peniadenisteroid A**

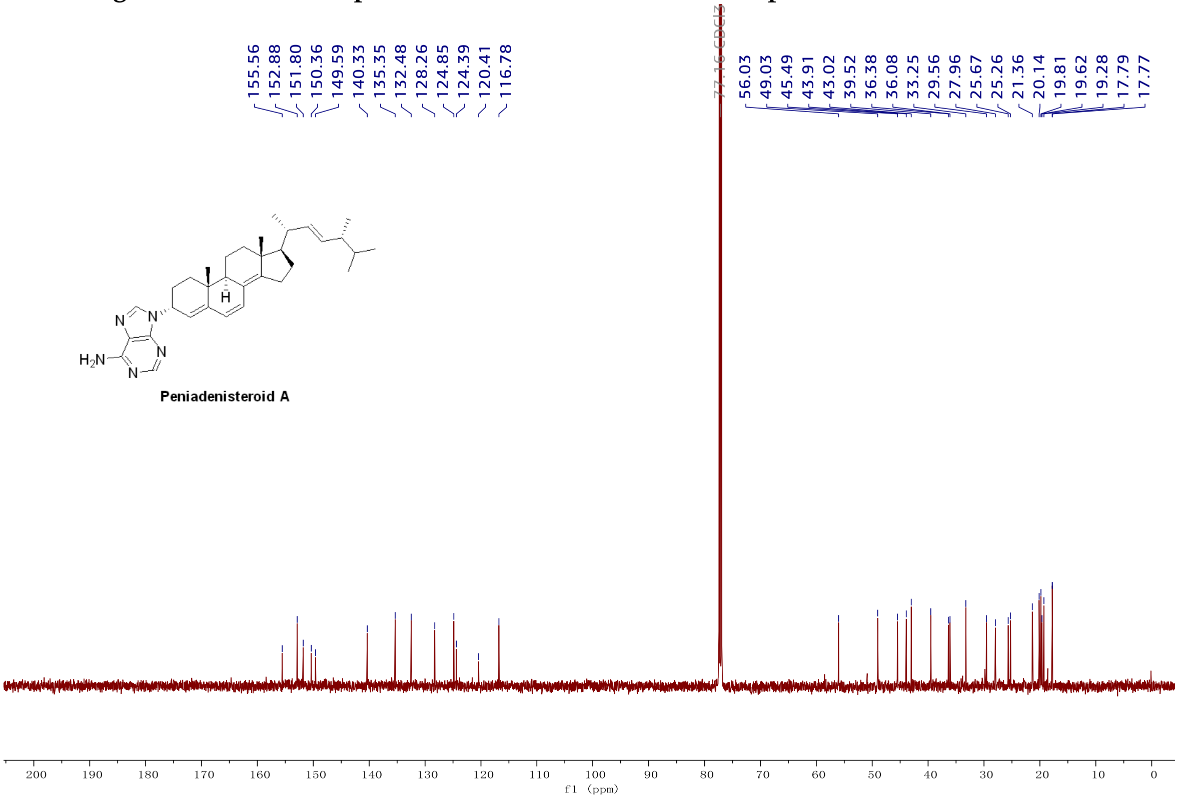

Figure S16. ^{13}C **NMR spectrum (150 MHz,** $CDCl_3$**) of compound Peniadenisteroid A**

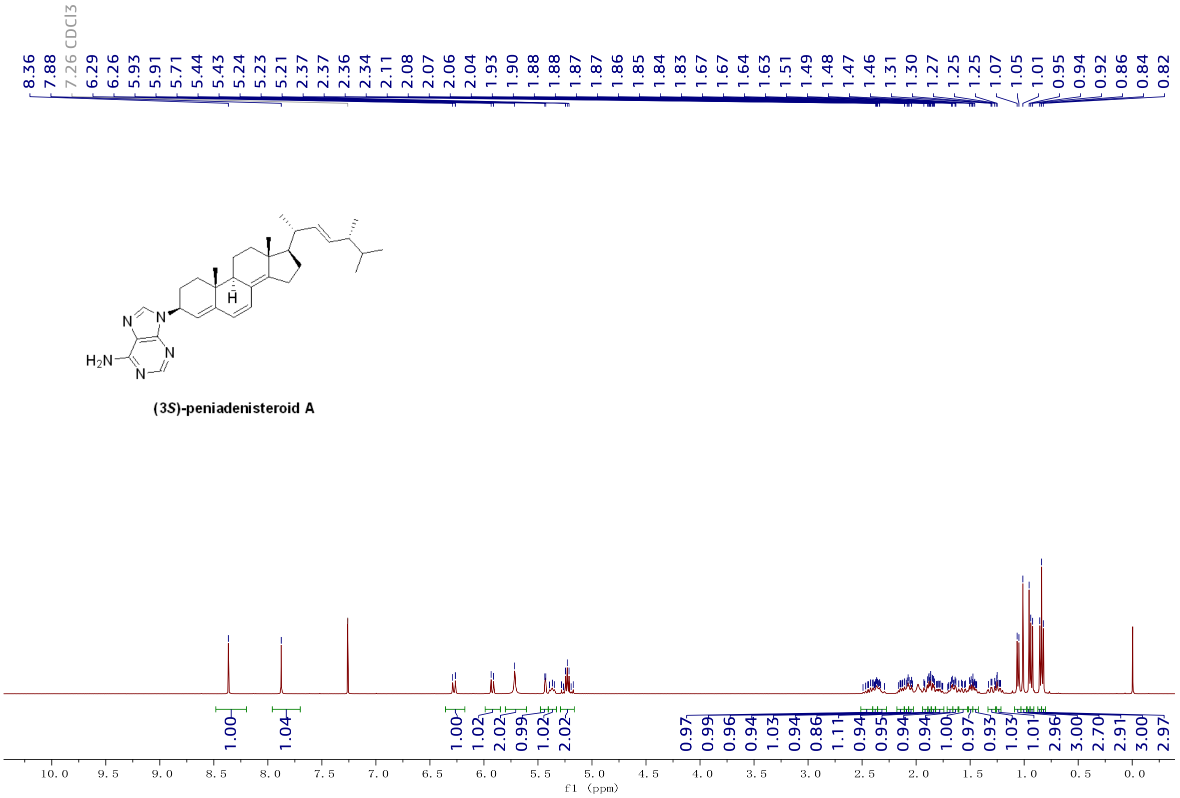

Figure S17. ^{1}H **NMR spectrum (400 MHz,** $CDCl_3$**) of compound (3*S*)-Peniadenisteroid A**

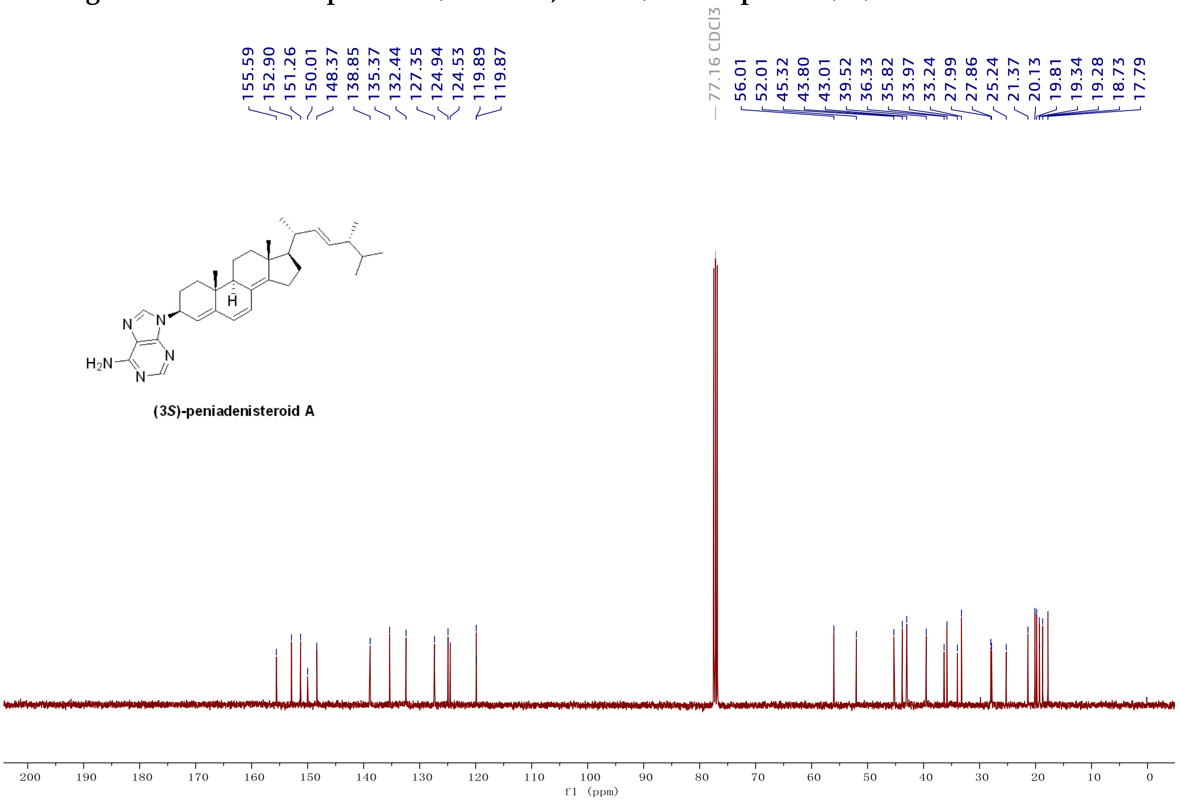

Figure S18. ^{13}C **NMR spectrum (100 MHz,** $CDCl_3$**) of compound (3*S*)-Peniadenisteroid A**

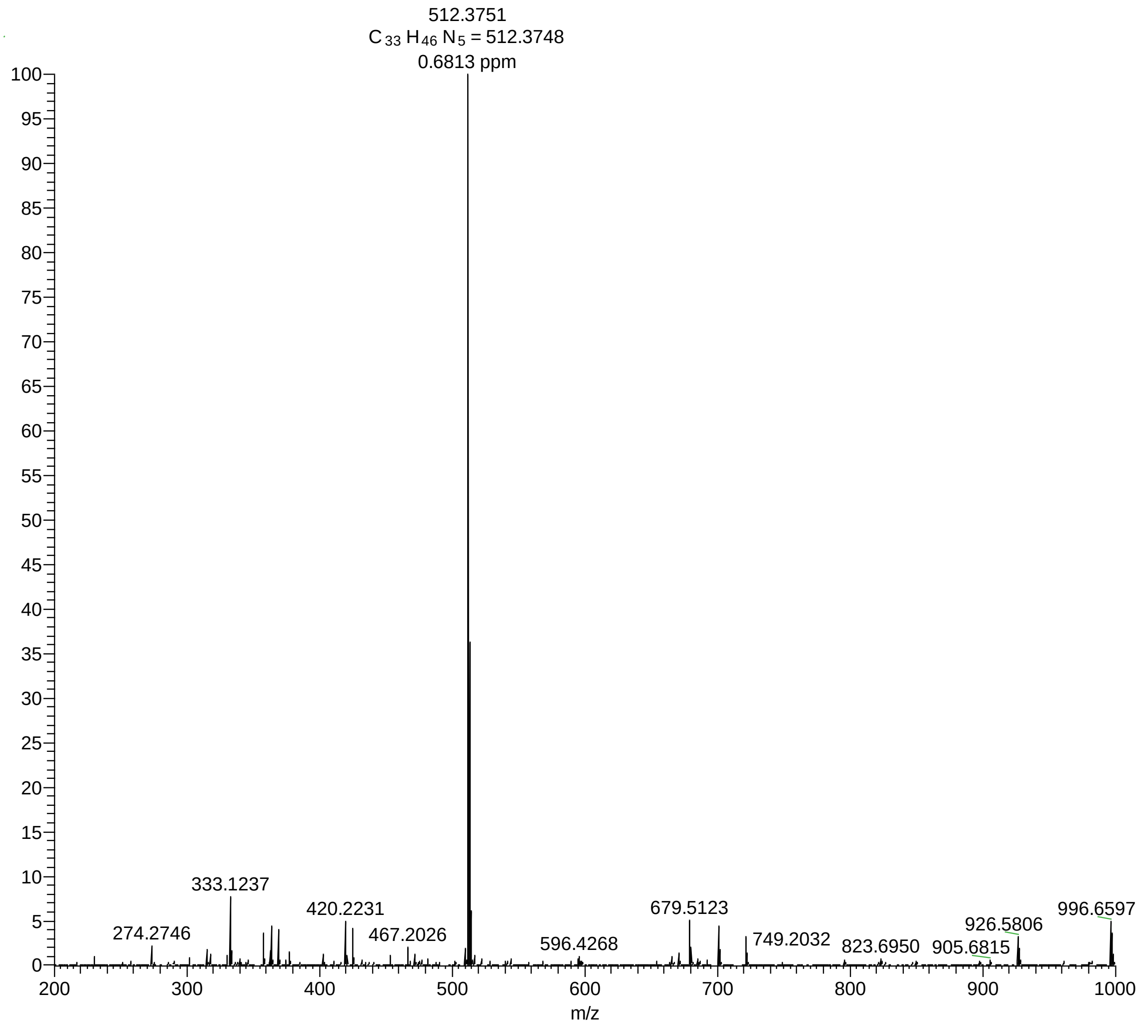

Figure S19. HRMS-ESI spectrum of compound (3*S*)-Peniadenisteroid A

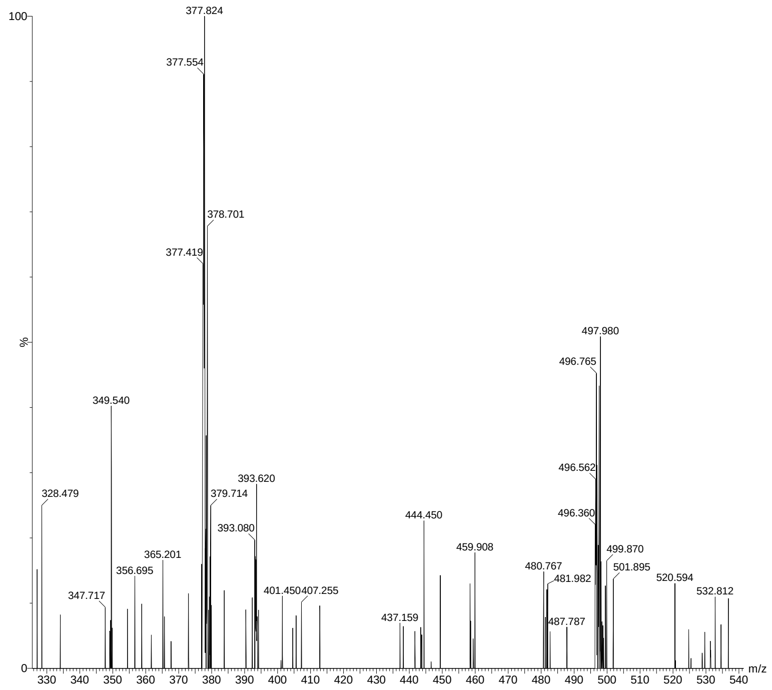

Figure S20. LRMS-ESI spectrum of compound P5a

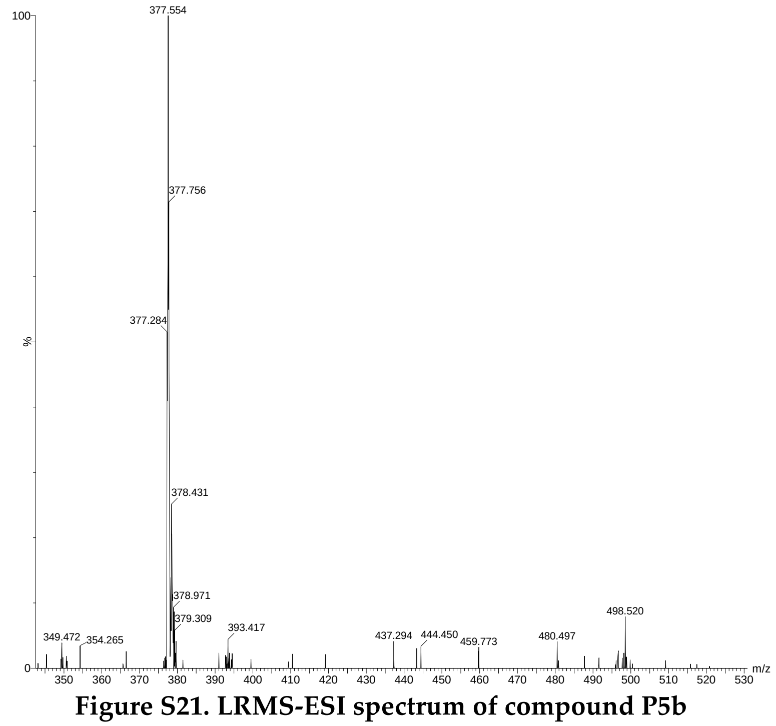

Figure S21. LRMS-ESI spectrum of compound P5b

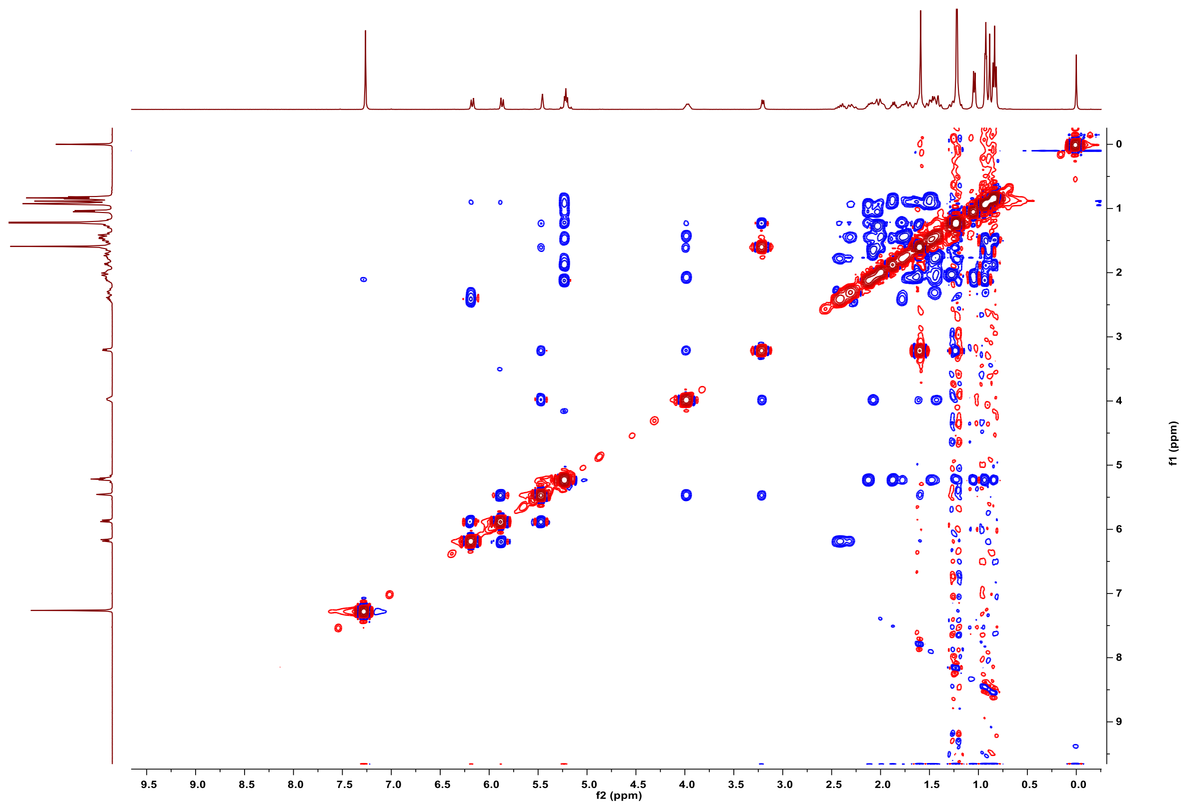

Figure S22. 2D NOESY spectrum of compound P5a

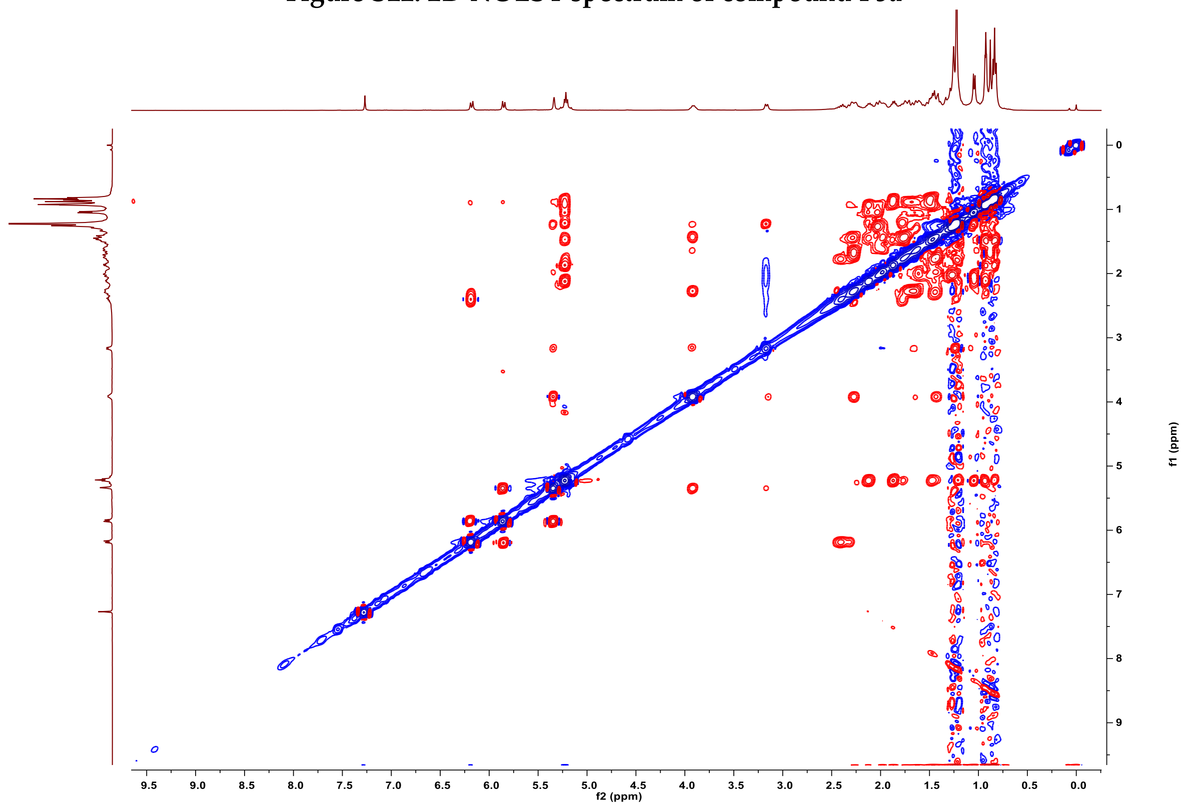

Figure S23. 2D NOESY spectrum of compound P5b

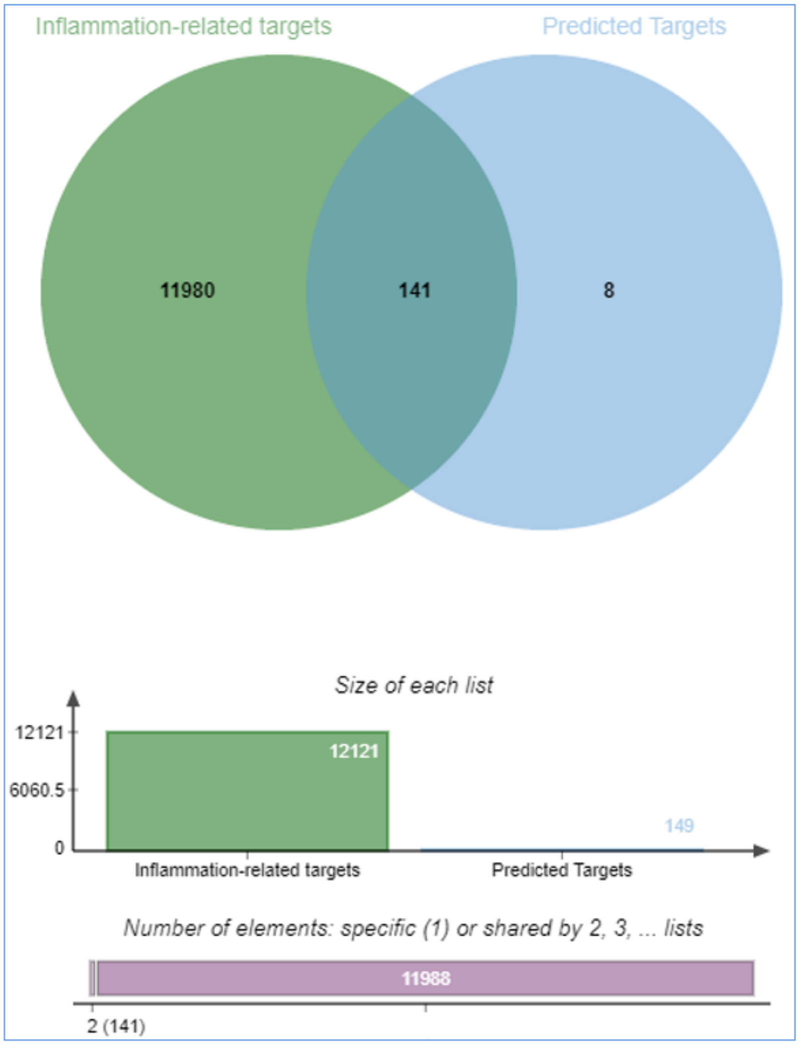

Figure S24. Venn diagram of overlapping targets between GeneCards and SuperPRED for (3*S*)-P6.
